# Proteomic analysis of phytase transgenic and non-transgenic maize seeds

**DOI:** 10.1038/s41598-017-09557-8

**Published:** 2017-08-23

**Authors:** Yanhua Tan, Zheng Tong, Qian Yang, Yong Sun, Xiang Jin, Cunzhi Peng, Anping Guo, Xuchu Wang

**Affiliations:** 1 Key Laboratory of Biology and Genetic Resources for Tropical Crops, Institute of Tropical Biosciences and Biotechnology, Chinese Academy of Tropical Agricultural Sciences, Haikou, Hainan 571101 China; 2 College of Agriculture, Hainan University, Haikou, Hainan 570228 China

## Abstract

Proteomics has become a powerful technique for investigating unintended effects in genetically modified crops. In this study, we performed a comparative proteomics of the seeds of phytase-transgenic (PT) and non-transgenic (NT) maize using 2-DE and iTRAQ techniques. A total of 148 differentially expressed proteins (DEPs), including 106 down-regulated and 42 up-regulated proteins in PT, were identified. Of these proteins, 32 were identified through 2-DE and 116 were generated by iTRAQ. It is noteworthy that only three proteins could be detected *via* both iTRAQ and 2-DE, and most of the identified DEPs were not newly produced proteins but proteins with altered abundance. These results indicated that many DEPs could be detected in the proteome of PT maize seeds and the corresponding wild type after overexpression of the target gene, but the changes in these proteins were not substantial. Functional classification revealed many DEPs involved in posttranscriptional modifications and some ribosomal proteins and heat-shock proteins that may generate adaptive effects in response to the insertion of exogenous genes. Protein-protein interaction analysis demonstrated that the detected interacting proteins were mainly ribosomal proteins and heat-shock proteins. Our data provided new information on such unintended effects through a proteomic analysis of maize seeds.

## Introduction

Genetically modified crops (GMCs) have been globally commercialized since 1996, and the cumulative planting area of GMCs has reached an unprecedented 2 billion hectares globally in 2015. A total of 28 countries allow planting of GMCs^1^. Although GMCs have delivered substantial benefits to farmers and our society, it is still debated whether GMCs should be widely accepted in many countries, including China, because of their unintended, unexpected and uncontrolled negative effects^2^. Therefore, determining the potential unintended effects of GMCs is important to understand the benefits of these crops to farmers and consumers.

Proteomics (protein profiling) is a powerful omics-based technique for investigating unintended effects at the protein level^2^. Proteomics is increasingly used because of its accuracy in describing protein diversity^3, 4^. Many proteins cannot be determined based only on the genes encoding their sequences, due to splicing events, enzymatic processing, or posttranslational modifications^5^. Traditionally, the high-resolution two-dimensional polyacrylamide gel electrophoresis (2-DE) technique has been used to detect potential unintended effects in proteomics research^6–10^. Recently, a non-gel-based protein analysis technique referred to as isobaric tags for relative and absolute quantification (iTRAQ) has been proposed for the quantitation of protein species and comparisons among samples. iTRAQ is a comparative proteomic approach that employs two-dimensional liquid chromatography (2D-LC) coupled directly with tandem MS (MS/MS) detection^11–13^. iTRAQ is considered more accurate and reliable than traditional 2-DE gel-based analysis^14–16^ and is thus now widely used for comparative proteomic analyses.

Maize is an important crop used for both food and feed, and transgenic maize has attracted much attention worldwide. Researches on commercialization of transgenic maize in China have made important potential contributions to maize production for meeting both national and global food and feed needs. Although maize is imported into China in large quantities (3.3 million tons in 2015), domestic production of transgenic maize has not yet been implemented to date. Many efforts have been devoted to transgenic maize, including Bt maize, herbicide-tolerant maize and phytase maize, but phytase-transgenic (PT) maize, which shows great potential in the animal feed industry, is the only genetically modified maize variety that has been officially issued a biosafety certificate in China^17^. PT maize was produced by achieving seed-specific overexpression of *Aspergillus niger* phytase (*phy*A2), an enzyme catalyzing the release of phosphate from phytase, which improves phosphorus availability and reduces the effect of animal production on the environment^18^. Many studies have been performed to ensure the safety of PT maize, including the investigation of its nutritional value^19^, used as livestock feed^20^, and the associated arthropod communities in the field^21^. We also performed a comparative proteomic analysis of leaves from PT maize and the non-transgenic (NT) isogenic variety^22^. However, any untargeted effects on the seeds of PT maize have yet to be studied through a proteomic analysis.

In the present study, we used traditional gel-based 2-DE and newly developed gel-free iTRAQ approaches to compare the quantitative differences in the proteomes of maize seeds between PT maize and the NT isogenic variety to investigate any unintended effects in PT maize seeds.

## Results

### Identification of Exogenous Genes and the Target Protein in PT Maize

**Figure 1:**
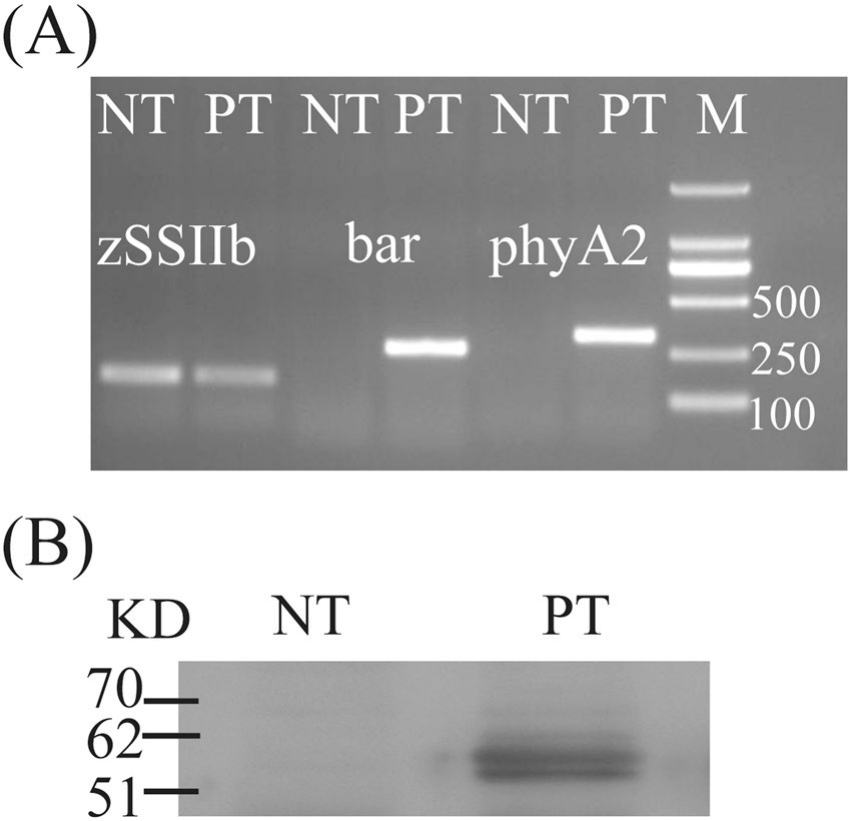
Detection of the exogenous gene expression and target proteins in maize seeds. (**A**) RT-PCR results for the exogenous genes in maize seeds; (**B**) Western blot analysis of target proteins in maize seeds.

The expression of exogenous genes was detected *via* semi-quantitative RT-PCR. The results presented in Fig. 1A show that the *bar* and *phy*A2 transcripts were detected in PT maize seeds. Neither the *phy*A2 nor *bar* transcript was detected in the NT control seeds of LIYU16. These results suggested that these exogenous genes were successfully expressed in PT maize seeds.

Western blotting analysis revealed a band of approximately 60 kDa in the PT maize seed samples (Fig. 1B), whereas the target band was not observed in the NT samples, indicating that the *phy*A2 protein accumulated only in these PT seeds.

### Comparison of Protein Profiles on 2-DE Gels

**Figure 2:**
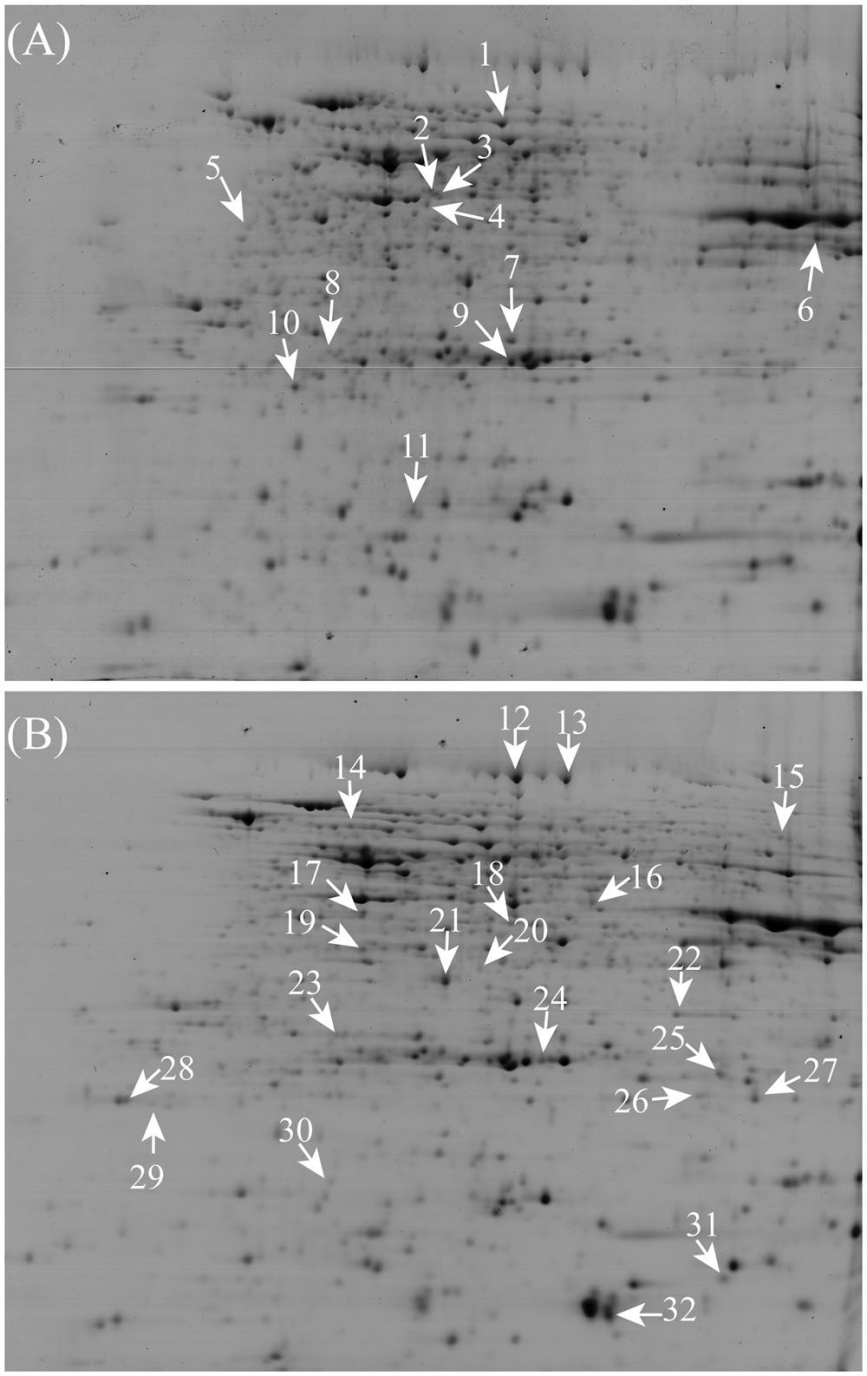
Typical 2-DE gels of total proteins from maize seeds. A total of 32 identified DEPs between PT and NT maize seeds were indicated with arrows in the gel images. The 11 DEPs with increased abundance (**A**) and 21 down-regulated DEPs (**B**) in PT maize seeds were highlighted in the 2-DE gels. The full-length original 2-DE gels were also presented in Supplementary Figure S1.

To compare the protein profiles of PT maize seeds and the corresponding wild-type variety, 2-DE maps of total proteins were obtained (Fig. 2 and Figure S1). Each sample was produced from three biological replicates, and for each biological replicate of the protein extracts, triplicate 2-DE gels were prepared as technical replicates. An average of 852 ± 94 spots were detected in the nine NT maize seed gels, while 826 ± 102 spots were detected in each of the nine PT maize seed gels, and there were 660 matching spots between the NT and PT maize seed gels (Fig. 2). Only spots showing a change >1.5-fold that were detected in all replicates were considered as differentially expressed proteins (DEPs). Analysis of the 2-DE images revealed 43 DEPs between the PT and NT maize seed samples (18 with higher and 25 with lower abundance compared with NT).

### Protein Identification *via* MALDI TOF/TOF MS

A total of 43 DEPs were selected for MALDI TOF/TOF MS analysis after in-gel digestion with trypsin^23^, and 32 protein spots were successfully identified through MS/MS analysis. Among these positively identified protein spots, 11 were up-regulated, and 21 were down-regulated, as shown in Fig. 2. Protein identification was based on homology to *Zea mays* proteins. Several spots were identified as containing more than one protein *via* MS/MS, in which the protein with the highest score that matched several members of grouped proteins in maize was chosen for further functional analysis (Table S1). The proteins that were indicated as uncharacterized were subjected to BlastP (protein-protein Blast) analysis in NCBI (http://blast. ncbi.nlm.nih.gov/ Blast.cgi) to determine their protein identities.

### Identification of Proteins in PT and NT maize seeds *via* iTRAQ

After merging data from the four biological replicates, a total of 300,833 spectra were generated from two independent iTRAQ experiments. Mascot search results identified a total of 73,765 distinct peptides and 6,313 detectable proteins from maize.

**Figure 3:**
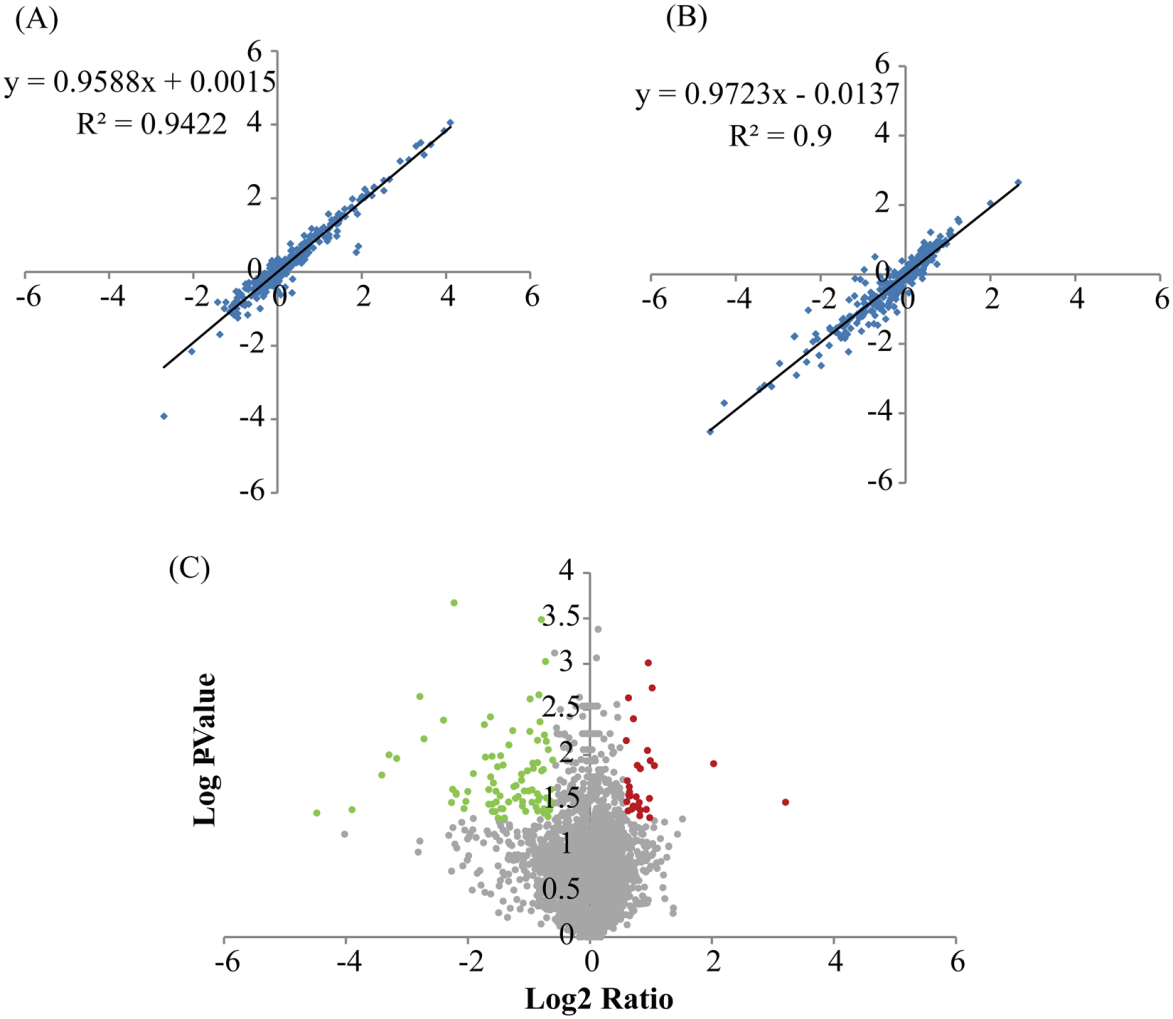
Comparison of log_2_ ratios and the volcano plot for the identified proteins from iTRAQ. Log_2_ values for the ratios of 114:115 in NT (**A**) and 116:117 ratios in PT (**B**) were used to show good replication in the iTRAQ experiments. The volcano plot shows the collected p-values (**C**) for the changes in the patterns of all identified proteins. Red spots represent up-regulated DEPs; gray spots are unchanged proteins; and green spots are down-regulated DEPs.

An analysis of variance of the data from two independent iTRAQ replicates was conducted to assess quantitative variations and reproducibility (Fig. 3A,B). The 114 and 115 tags indicate the two NT maize replicates, and 116 and 117 tags indicate the two PT maize replicates in each iTRAQ experiment. Thus, we compared the log_2_ iTRAQ ratios of 114:115 and 116:117 (Fig. 3). Plots of comparable quantification results for the 114:115 and 116:117 ratios were determined *via* linear regression analysis. The results showed a slope of ≈0.96 at an R^2^ of 0.94 for 114:115 and ≈0.97 at an R^2^ of 0.90 for 116:117, suggesting that the quantitative iTRAQ data from replicates were reproducible^24, 25^.

### Determination of DEPs by iTRAQ from the PT and NT maize seeds

A total of 6313 non-redundant proteins were detected *via* iTRAQ under a false discovery rate (FDR) of less than 1%. Among all identified proteins, 116 DEPs between PT and NT maize seeds were obtained based on the following criteria: confident peptides ≥2, error factor <2, change ratio >1.5 and P-values <0.05. Among the 116 DEPs, 31 proteins exhibited an increase of more than 1.5-fold in PT maize seeds, while 81 proteins exhibited a decrease to less than 0.667-fold in the PT maize seeds compared with NT seeds (Fig. 3C and Table S2).

These results, combined with those of 2-DE, showed a total of 148 identified DEPs, 42 of which showed higher expression, while 106 showed lower expression in PT than in NT.

### Bioinformatics Analysis of DEPs Identified in iTRAQ and 2D Gels

Bioinformatics analysis was performed for all 148 DEPs identified through 2-DE and iTRAQ analyses.

**Figure 4:**
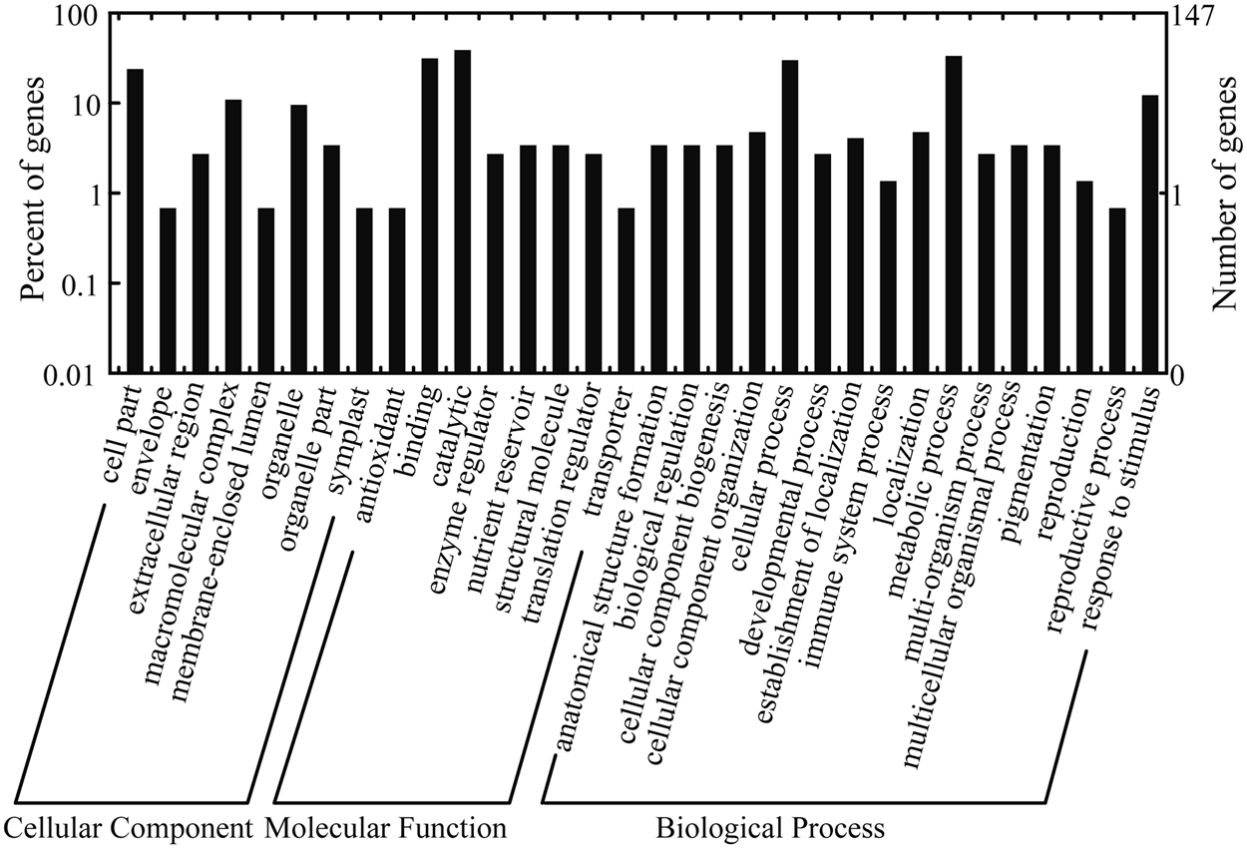
GO annotation of DEPs in PT and NT maize seeds.

To identify the significantly enriched GO functional groups of DEPs between NT and PT maize seeds, GO annotation was performed using WEGO software (http://wego.genomics.org.cn/cgi-bin/wego/index.pl). GO information was obtained with BLAST2GO 3.0. The results showed that 141 DEPs were successfully mapped with GO annotations, which were classified into three ontologies containing 32 functional groups (Fig. 4 and Table S3). At the cellular GO level, 8 GO terms were obtained, corresponding to 35 proteins (23.8%) in the cell part category (GO:00044464), and 16 proteins (10.9%) in the macromolecular complex category (GO:0032991). For the molecular function ontology, 8 GO terms were found; the major functional groups were catalytic activity (GO:0003824), with 57 proteins (38.8%), and binding functional groups (GO:0005488), with 46 proteins (31.3%). In the biological process category, 16 GO terms were assigned. The major functional groups of the DEPs were involved in metabolic processes (GO:0008152), including 49 proteins (33.3%), followed by cellular processes (GO:0009987), including 44 proteins (29.9%).

**Figure 5:**
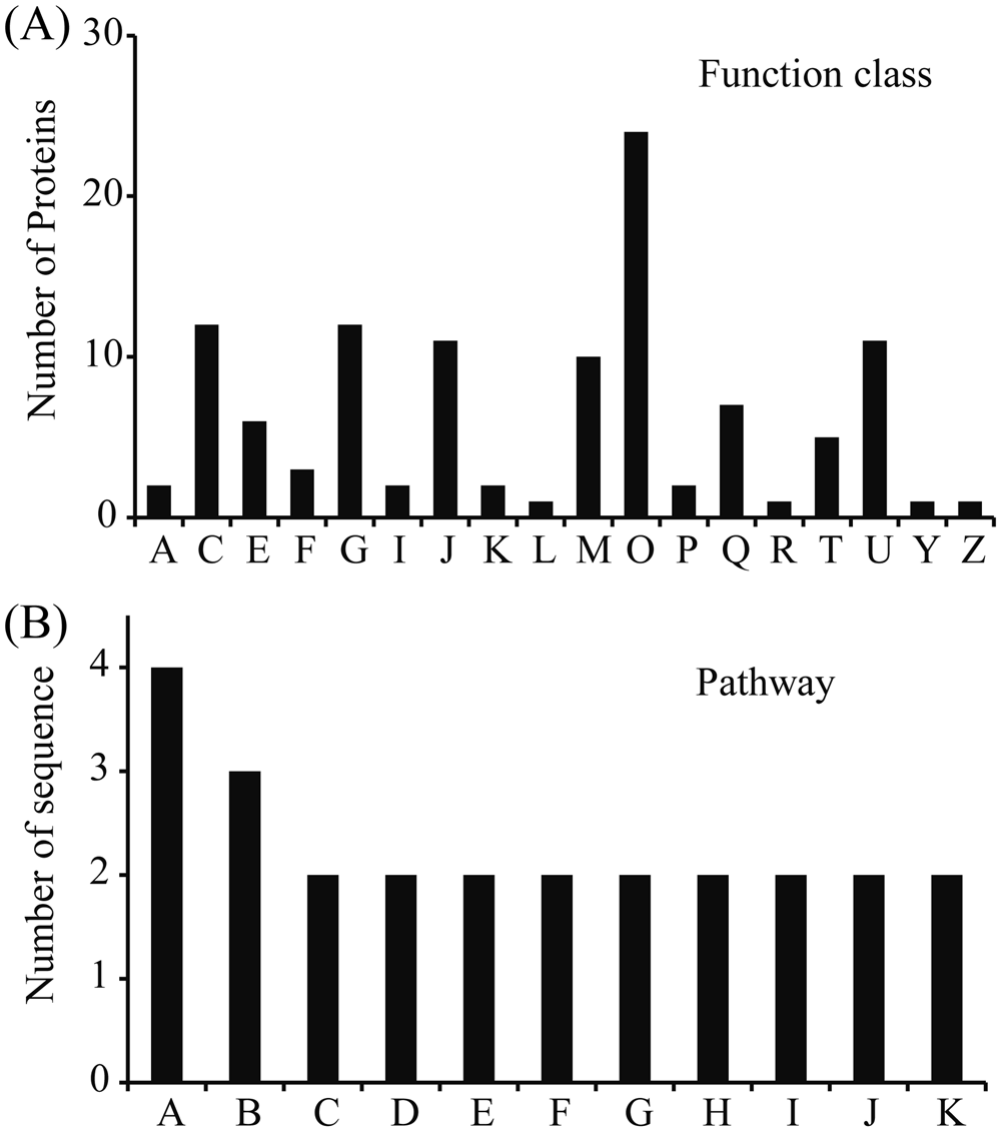
COG classification and KEGG pathway analysis of DEPs between PT and NT maize seeds. Functional class was produced by COG (**A**), and the abbreviations in the figure (**A**) are as follows: A, RNA processing and modification; C, Energy production and conversion; E, Amino acid transport and metabolism, Coenzyme transport; F, Nucleotide transport and metabolism; G, Carbohydrate transport and metabolism; I, Lipid transport and metabolism; J, Translation, ribosomal structure and biogenesis; K, Transcription; L, Transcription, Replication, recombination and repair; M, Cell wall/membrane/envelope biogenesis; O, Post-translational modification, protein turnover, and chaperones; P, Inorganic ion transport and metabolism; Q, Secondary metabolites biosynthesis, transport, and catabolism; R, General function prediction only; T, Signal transduction mechanisms; U, Intracellular trafficking, secretion, and vesicular transport; Y, Nuclear structure; Z, Cytoskeleton. The related KEGG pathways were classified into 11 main categories (**B**), and the abbreviations in the figure (**B**) are as follows: A, Amino sugar and nucleotide sugar metabolism; B, Pyruvate metabolism; C, Starch and sucrose metabolism; D, Galactose metabolism; E, Metabolism of xenobiotics by cytochrome P450; F, Histidine metabolism; G, Carbon fixation in photosynthetic organisms; H, Glutathione metabolism; I, Drug metabolism - cytochrome P450; J, Aminobenzoate degradation; K, Pentose and glucuronate interconversions.

The categories of the DEPs between NT and PT maize seeds were further grouped according to their primary biological activities, as defined by the functional catalogue of Clusters of Orthologous Groups (COG) of proteins. The results showed that 131 DEPs (76.4%) were classified into 18 COG categories, among which, “posttranslational modification, protein turnover, chaperones” represented the largest group (group O, 24 DEPs), followed by “energy production and conversion” (group C, 12 DEPs) and “carbohydrate transport and metabolism” (group G, 12 DEPs). Approximately 10% of the DEPs were related to “translation, ribosomal structure and biogenesis” (group J, 11 DEPs) and “intracellular trafficking, secretion, and vesicular transport” (group U, 11 DEPs) (Fig. 5A and Table S4).

To further investigate the biological functions of these DEPs, KEGG pathway analysis was performed using the BLAST2GO 3.0 program. A total of 77 DEPs (52%) were mapped to 37 pathways in the KEGG database. The most represented pathway was “amino sugar and nucleotide sugar metabolism,” which contained four sequences (Spots 52, 71, 96, and 124). The other major pathway was “pyruvate metabolism” which contained three sequences (Spots 52, 64, and 113). Two DEPs were involved in each of the following pathways: “starch and sucrose metabolism,” “galactose metabolism,” “metabolism of xenobiotics by cytochrome P450,” “histidine metabolism,” “carbon fixation in photosynthetic organisms,” “glutathione metabolism,” “drug metabolism - cytochrome P450,” “aminobenzoate degradation,” and “pentose and glucuronate interconversions”. The remaining pathways contained only one sequence each (Fig. 5B and Table S5).

**Figure 6:**
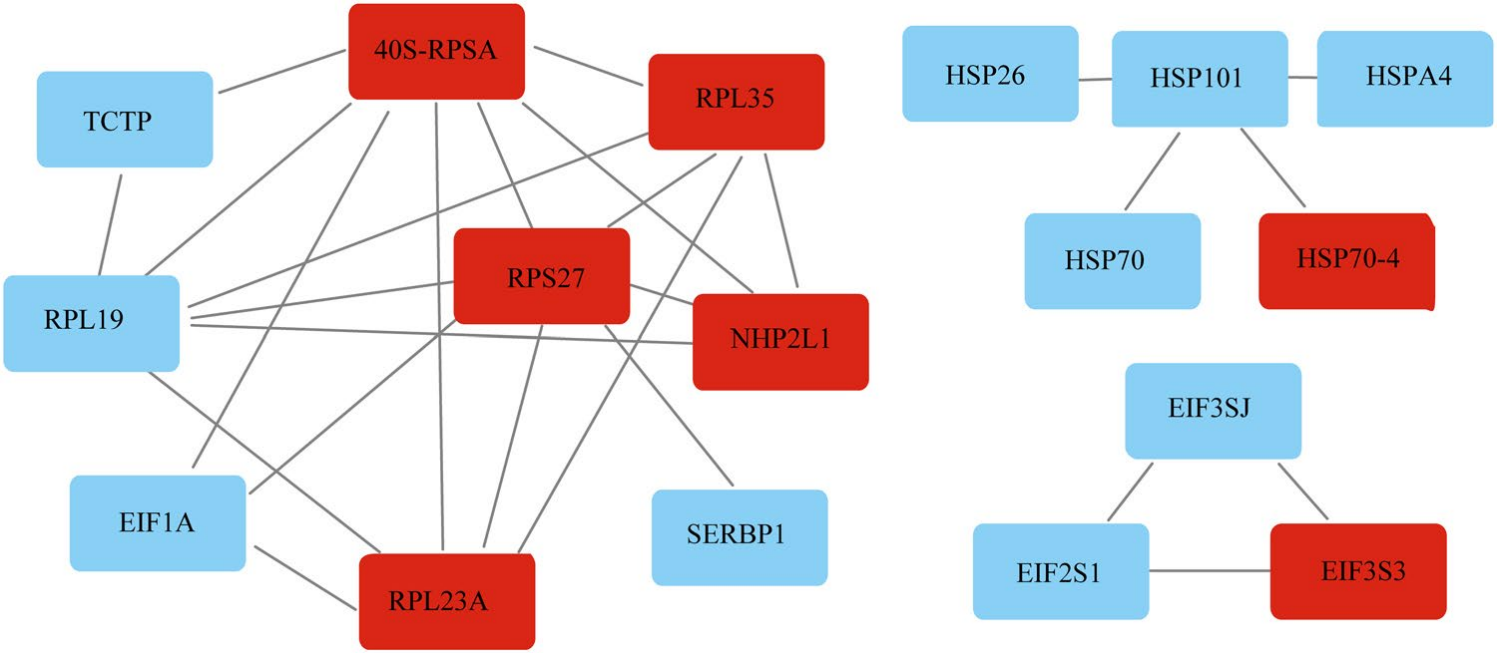
Protein-protein interaction analysis of DEPs. A total of 17 proteins were involved in protein-protein interactions that contained 3 main clusters with 7 up-regulated (marked with red blocks) and 10 down-regulated proteins (blue blocks).

To identify protein-protein interaction networks, the identified 148 DEPs were subjected to STRING (v10) analysis and visualized by Cytoscape 3.40 software with high confidence. In the 148 proteins, 17 were involved in protein-protein interactions, which were included in three main clusters, with seven up-regulated and 10 down-regulated proteins (Fig. 6). Among these proteins, eight interacting proteins were mainly related to “translation, ribosomal structure and biogenesis,” while five interacting proteins were related to “post-translational modification, protein turnover, and chaperones” among the COG categories. The 40S ribosomal proteins SA (40S-RPSA) and S27 (RPS27) were found to be the central core proteins in these interacting networks, associating with seven other proteins. The other core proteins were the ribosomal protein L19 and the 60S ribosomal protein L35, which interacted with six other proteins, and a 60S ribosomal protein knows as L23a, which interacted with five other proteins.

### Comparison of Protein and Transcript Expression Patterns

**Figure 7:**
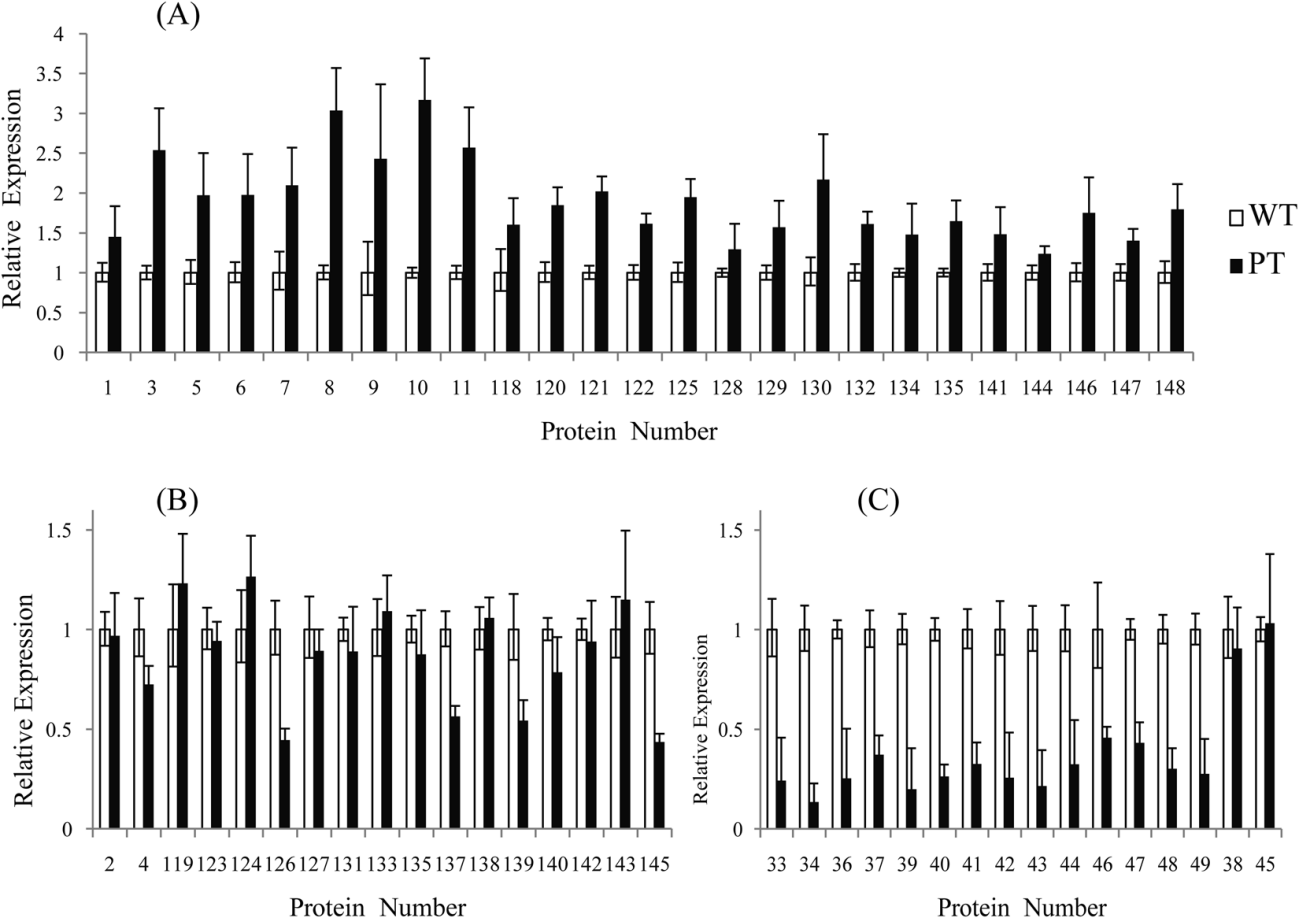
qRT-PCR analysis of the gene expression patterns of the 58 identified DEPs. Among the 42 up-regulated DEPs, the transcript levels of 25 genes displayed the same trend, with increased abundance of the corresponding protein species (**A**), while the results for 17 genes were inconsistent with observed translation levels (**B**); among the 16 down-regulated DEPs, 14 genes exhibited similar change trends, and two genes showed no significant difference at the transcript level (**C**).

To explore the correspondence between the level of mRNA transcripts and the abundance of accumulated proteins, transcriptional analysis of 58 selected DEPs, including 42 up-regulated and 16 down-regulated protein species was performed *via* qRT-PCR. Most of the genes exhibited a similar change trend at the transcriptional and translational levels (Fig. 7). Among the 42 up-regulated DEPs, the transcript levels of 25 genes displayed the same change trend as the abundance of the corresponding protein species. Additionally, 12 genes showed no difference at the transcript level and five genes exhibited the opposite trend from the observed translation levels. The five inconsistent proteins were pro-resilin, MIR-interacting saposin-like protein, xyloglucan endotransglucosylase/hydrolase, activator of 90 kDa heat-shock protein ATPase and an uncharacterized protein. Among the 16 down-regulated DEPs, 14 genes exhibited a similar change trend and two genes showed no difference at the transcript level. The discrepancy between the transcription and translational levels probably resulted from the presence of various posttranslational modifications^26^ or the higher sensitivity of iTRAQ compared with that of qRT-PCR^27^.

## Discussion

The 2-DE method has been a commonly applied separation technique for decades in proteomic studies^28^. However, as a separation method for proteomic analyses, 2-DE presents several limitations^29^. The routine 2-DE approach allows the detection of a limited number of protein spots compared with iTRAQ and other high-throughput quantitative proteomic methods^30^. It is considered that 2D PAGE can resolve up to only 5000 protein spots simultaneously, depending on the gel size and pH gradient used^31^. Moreover, hydrophobic proteins, proteins with a very high or very low molecular weight, and highly acidic or highly basic proteins are often lost during 2-DE gel separation^29^. However, 2-DE is especially suitable for visual detection of high abundant proteins and their isoforms^32^.

The iTRAQ method is a gel-free method for shotgun quantitative analysis. It is a second-generation proteomic technique with high sensitivity, excellent accuracy and high throughput. Proteins with low quantity or that are difficult to separate *via* 2-DE can be analyzed by iTRAQ. However, iTRAQ cannot detect many high abundant proteins that can be detected by 2-DE; this inconsistency is clear in the comparison of proteomic studies on maize (*Zea mays*) under salt and drought stress performed using 2-DE and iTRAQ approaches^32–36^. The partial overlap between the outputs of 2-DE and iTRAQ approaches is limited, due to their different characteristics^32, 37^.

In the present study, we used 2-DE- and iTRAQ-based quantitative proteomic methods to compare the proteomic characteristics of the seed between PT maize and its NT isogenic counterpart. By taking advantages of these two quantitative techniques, we performed an in-depth study and obtained much new information. In our study, only 43 DEPs were observed in 2D gel, among which 32 proteins were successfully identified, whereas 6313 proteins were successfully identified *via* iTRAQ, 116 of which (1.83%) were considered as DEPs. However, only a few of the proteins identified *via* 2-DE overlapped with the iTRAQ DEPs. This discrepancy might be related to many factors, such as protein physicochemical properties, peptide preparation, quantification way, and proteins with post translational modifications. We consider that these two methods are complementary and multiple strategies are required to obtain high proteomics data. Therefore, the outputs of the two methods should be merged for the subsequent analysis.

A new GMC may exhibit potential unintended effects due to the random insertion of exogenous genes^2^. To assess the effects caused by *phy*A2 gene insertion, a comparative proteomic analysis of the seeds between PT maize and its NT isogenic counterpart was conducted. Using 2-DE- and iTRAQ-based quantitative proteomics techniques, approximately 148 identified DEPs were detected. Compared with the total number of proteins identified using iTRAQ (6313), the number of DEPs identified was significantly smaller, and a limited number of proteins were found to be affected by gene insertion^38^. Moreover, the DEPs identified in the transgenic plants were not identified as new proteins but as proteins showing changes in abundance. These results agreed with our findings in maize leaves^22^ and the findings of many other studies^39, 40^, indicating that DEPs were detectable but showed no substantial differences in the seeds of PT versus NT maize after overexpression of target genes^8, 22, 41^.

Unfortunately, we did not detect the target phytase protein, encoded by the *phy*A2 gene, as a DEP. The DEPs identified using the 2-DE approach may have been due to low expression of the target gene and the limited accumulation of the target protein in PT seeds, as described previously^22^. Regarding the DEPs obtained by iTRAQ, which is a comparative quantitative proteomic analysis method, it must be considered that iTRAQ shows considerable deficits in differential protein detection^30^.

COG functional classification showed that approximately 20% of DEPs were associated with “posttranslational modification, protein turnover, chaperones” and 11% with “translation, ribosomal structure and biogenesis”. Therefore, approximately 31% of the DEPs were involved in posttranscriptional regulation and posttranscriptional modifications in PT maize seeds. Regarding the function of posttranscriptional regulation, most of the related DEPs were ribosomal proteins. In the nucleolus, ribosomal proteins form the mature 40S and 60S subunits of the mature ribosome (the site of protein synthesis), which is a process of fundamental importance in cell biology^42^. In the present study, the abundance of most ribosomal proteins was found to increase, and changes in mRNA expression levels were up-regulated in PT maize seeds. These up-regulated effects might be a response to the insertion of exogenous genes.

In terms of posttranscriptional modifications, most of the related DEPs were heat shock-proteins. HSPs promote the degradation of abnormal proteins, functioning as “molecular chaperones” to prevent the aggregation of denatured proteins and promote the proper refolding of these proteins^43, 44^. Our results showed that most HSP70s were up-regulated in PT maize seeds. Conversely, the abundance of HSP26 and other chaperonin family proteins was decreased in PT maize seeds (Table S4); this finding suggested that different members of HSPs exhibit different functions in response to the insertion of exogenous genes.

Protein-protein interaction analysis also showed that most interacting proteins were associated with “posttranslational modification, protein turnover, chaperones” and “translation, ribosomal structure and biogenesis,” and those interacting proteins were mainly ribosomal proteins and heat-shock proteins.

KEGG analysis revealed that only 52% of the DEPs could be mapped to the 37 pathways, and only 3 up-regulated DEPs could be mapped to 5 pathways. In these pathways, most of the enzymes were dehydrogenases, which were involved in 20 pathways, followed by phosphatases involved in 5 pathways. GO annotation also showed that the major functional groups were catalytic activity and binding functional groups in terms of molecular function ontology. Phytases are classified as phosphatases that catalyze the production of less-phosphorylated myo-inositol derivatives and inorganic phosphate from phytic acid^45^. These phosphatase signals may have occurred in response to the insertion of the *phy*A2 gene.

In conclusion, we conducted a comparative proteomic analysis to evaluate the unintended effects of PT maize seeds by combining 2-DE and iTRAQ approaches. A total of 148 DEPs were successfully identified between PT and NT maize seeds, among which 42 were up-regulated and 106 were down-regulated in PT maize seeds compared with NT maize seeds. Bioinformatics analysis showed that most of the DEPs were involved in posttranscriptional regulation and posttranscriptional modification functions in PT maize seeds. Ribosomal proteins and heat shock proteins might present adaptive effects in response to the insertion of exogenous genes. By taking advantages of the 2-DE and iTRAQ approaches, the data generated in the present study provided more information on such unintended effects than the data from target-oriented analyses. However, the number of proteins that could be analyzed was still limited. Unintended effects are widely observed during conventional plant breeding and are not unique to GM plants. Biosafety assessment of GM plants should be performed in a case-by-case manner. Our proteomics data for PT maize seeds may be beneficial for the commercialization of PT maize and will provide much more information for the biosafety assessment of GMCs in the future.

## Materials and Methods

### Plant Materials

The transgenic maize variety 10TPY006 is a phytase gene-overexpressing variety (PT maize), and the corresponding non-genetically modified control is the conventional hybrid LIYU16 (NT maize). LIYU16 planted in China is commonly used as feed for animals. The genetic background of the two plant materials was described in our previous study^22^.

Seeds of PT and NT maize were provided by the Beijing Origin Seed Technology Inc. The PT and NT seeds were planted in a greenhouse, and each line was planted in three micro-plots as three replicates. After sowing, the plants were treated with the following standard agricultural practices. The ears were harvested after physiological maturity. Ears of three plants from each micro-plot were harvested at the same time on the same day, and immediately stored at −80 °C for further study. For the following analysis, seeds derived from the central position of each ear were ground into fine powders.

### RT-PCR and Weston Blot Detection of the Target Protein

Semi-quantitative RT-PCR was performed to examine the expression of exogenous genes in transgenic maize. Total RNA was isolated from seeds by the TRIzol method using the TIRpure reagent (Bioteke, Beijing, China). The *zSSIIb* gene was amplified using the *zSSIIb*-F (5′-CGGTGGATGCTAAGGCTGATG-3′) and *zSSIIb*-R (5′-AAAGGGCCAGGTTCATTATCCTC-3′) primers as an internal control. The *bar*-F (5′-GAAGGCACGCAACGCCTACGA-3′) and *bar*-R (5′-CCAGAAACCCACGTCATGCCA-3′) primers were employed for the *bar* gene, and the *phy*A2 event-specific primers used for RT-PCR were P-F (5′-TCAAACCCTTCACGAAGCTATCCC-3′) and P-R (5′-TACTTTCCCGCTCAACTCCACTCT-3′) as described previously^22^. Reference materials were provided by Beijing Origin Seed Technology Inc. and were used as positive and negative controls.

Weston blot detection was performed as described previously^18^. Proteins were extracted from seeds using GLY-HCl buffer (0.05 mol/L, pH 3.0)^46^, and approximately 20 µg of the total protein in the supernatant was employed for SDS-PAGE. After separation on an SDS-PAGE gel, the proteins were transferred onto a polyvinylidene difluoride (PVDF) membrane (GE Healthcare) for Western blot analysis. The blot was probed with a polyclonal antibody for the phyA2 protein provided by the Biotechnology Institute, Chinese Academy of Agricultural Sciences (CAAS) (1:2000 dilution), and goat anti-rabbit IgG-labeled with horseradish peroxidase (HRP) was used as the secondary antibody.

### Protein Extraction from Seeds

Each sample contained three biological replicates. Total seed proteins were extracted using a modified Borax/PVPP/Phenol (BPP) protein extraction method as described^47^. Approximately 3 g of the frozen maize seeds were ground into fine powders in liquid nitrogen and resuspended in 10 mL of extraction buffer. After 5 min, an equal volume of Tris-saturated phenol (pH 8.0) was added into the suspension, and the mixtures were vortexed for 10 min. The mixtures were then centrifuged (16,000 g, 15 min, 4 °C), and the upper phase was transferred to a new centrifuge tube. Then protein precipitates were obtained by adding five volumes of ammonium sulfate saturated-methanol, followed by incubation at 22 °C for at least 6 h. The precipitated proteins were centrifuged as indicated above, and the protein pellet was resuspended and rinsed twice with ice-cold methanol and ice-cold acetone. Finally, the protein pellets were air-dried and recovered with lysis buffer to determine the protein concentration according to the Bradford method, using bovine serum albumin as a protein standard, or were stored at −20 °C.

### 2-DE Electrophoresis

Approximately 1,300 µg of the protein samples were diluted to a volume of 450 µL with lysis buffer (7 M urea, 2 M thiourea, 2% CHAPS, 13 mM DTT), followed by loading onto a 24-cm IPG strip (immobilized pH gradient) with a linear pH gradient of 4–7 (GE Healthcare, Uppsala, Sweden). The strips were hydrated for 18 h at room temperature, and then subjected to IEF on an Ettan IPGphor isoelectric focusing system, following the instructions of the manufacturer (2-DE Manual, GE Healthcare), with some modifications as described previously^22^. After IEF, the IPG strips were equilibrated first in an equilibration solution containing 1% DTT, followed by an equilibration solution with 4% iodoacetamide. The strips were then transferred to an Ettan Dalt system (GE Healthcare) to perform SDS-PAGE using 12.5% SDS polyacrylamide gels^47^.

The gels were visualized *via* the GAP staining method^48^ and scanned with ImageMaster Labscan V3.0 (GE Healthcare, Uppsala, Sweden), and image analysis was performed with the ImageMaster 2D Platinum software package (GE Healthcare, Uppsala, Sweden). Spots that were present in all replicate gels, showing Student’s *t* test p-values <0.05 and a relative fold change of at least a 1.5 in their quantity, were further analyzed.

### Protein Identification in 2-DE Gels *via* MALDI-TOF MS/MS

The target protein spots of DEPs were manually excised from the 2-DE gels and digested in-gel with bovine trypsin (Trypsin, Roche, Cat. 11418025001) as described previously^23^. Digested proteins were then mixed with alpha-cyano-4-hydroxycinnamic acid (CHCA) matrix for determination of mass spectra and analyzed using an AB SCIEX MALDI TOF-TOF 5800 system (AB SCIEX, Shanghai, China) equipped with a neodymium with laser wavelength 349 nm. Mass spectra were obtained as described previously^49^.

The raw MS and MS/MS spectra were combined and searched against the *Zea mays* amino acid sequence database (including 87,603 sequences), which was downloaded from Uniprot (http://www.uniprot.org), using in-house MASCOT software for protein identification. The searched parameters were set as previously described^22^. If peptides matched multiple proteins, the protein with the highest score was recorded in this study for bioinformatics analysis. For the unnamed proteins, BLAST search using NCBI (http: //www.ncbi.nlm) was performed to identify homologous proteins.

### iTRAQ Labeling, Fractionation and Nano-LC-MS/MS Analysis

Proteins from two biological replicate samples were used for quantitative proteomic analysis. The iTRAQ experiment was conducted on a Triple TOF 5600 system (AB Sciex) coupled with a Nanoflex microchip system (Eksigent, Dublin, USA) as described^50^. A total of 100 μg of proteins was collected from each sample solution and digested with trypsin (AB Sciex, No. 4352157). The digested peptides were labeled using iTRAQ 4-plex kits (Applied Biosystems, USA), following the instructions of the manufacturer. The NT maize replicates were labeled with the iTRAQ 114 and 115 tags, and the PT maize replicates were labeled with the 116 and 117 tags. The labeled peptide mixtures were pooled and then dried by vacuum centrifugation.

The labeled peptide mixtures were re-dissolved with buffer A (20 mM ammonium formate, pH 10.0) and fractioned using a C18 column (4.6 mm × 250 mm, 5 μm 100 Å, Agela). The peptides were eluted at a flow rate of 0.8 mL/min, and the gradient of fraction was set as follows: 5% buffer B (20 mM ammonium formate, pH 10.0, 80% CAN) for 0–5 min, 5–25% buffer B for 5–30 min, 25–40% buffer B for 30–45 min, 40–90% buffer B for 45–55 min, 90% buffer B, 55–64 min, and 90–5% buffer B for 64–65 min. The chromatogram was monitored through a UV detector, and UV wavelengths were set at 280 and 214 nm. Fractions were collected at 1 min intervals, with a total of 65 fractions being collected and lyophilized. The eluted peptides were pooled into 10 fractions for subsequent nano-LC-MS/MS analysis.

Peptides were eluted from the HPLC column *via* the application of a gradient from 5% buffer B (98% ACN v/v, 0.1% FA v/v) to 55% buffer B for 79 min, running at 300 nL/min, followed by a linear gradient to 80% buffer B in 1 min. The obtained peptides were subjected to analysis using a Triple TOF 5600 mass spectrometer, which was operated in an information-dependent data acquisition mode. The parameters were set as follows: ion spray voltage: 2.3 kV; GS1: 4; curtain gas: 30; DP: 100; m/z: 350–1250; accumulation time: 0.25 s; product ion scan: IDA number: 30; m/z: 100–1500; accumulation time: 0.1 s; dynamic exclusion time: 25 s; rolling CE: enabled; adjust CE when using iTRAQ reagent: enabled; CES: 5.

### iTRAQ Protein Identification and Quantification

Protein identification was performed using ProteinPilot^TM^ software (AB Sciex Inc., USA) against the *Zea mays* database (87,603 sequences) from the UniprotKB database. To evaluate the consistency among replicates, quantitative ratios of protein species abundance between the four biological replicates were compared with a value of 1. For relative protein quantification, one protein was required to contain at least two unique peptides. The quantitative protein ratios were weighed and normalized according to the median ratio in Mascot. Proteins showing a fold change of at least 1.5 and a P-value <0.05 were further analyzed in four biological replicates.

### DEPs Functional Classification and Pathway Analysis

Functional annotations of the DEPs identified through combined 2-DE and iTRAQ analyses were performed. COG analysis of the proteins was performed for functional classification *via* searches in a database (http://eggnogdb.embl.de/). GO classification according to GO terms was further performed online using WEGO software (http://wego.genomics.org.cn)^51^. In addition, KEGG pathway analysis was conducted to determine the reaction networks of all identified DEPs using Blast2GO 4.0 against the non-redundant protein database (NR; NCBI) (http://www.geneontology.org/). Finally, protein-protein interactions were analyzed using the STRING v10 database (http://string-db.org) for the identified proteins to determine the functions and pathways that were most strongly associated with the protein list.

### RNA Extraction and Quantitative Real-time PCR

Total RNA was extracted with the TRIzol reagent, and 1 μg of RNA was used for reverse transcription into cDNA with a reverse transcriptase kit (TaKaRa, Tokyo, Japan). The mixed solution of the qRT-PCR reaction with a 20 μL volume was prepared in triplicate by adding 1 μL of each cDNA dilution to SYBR Green PCR Master Mix (TaKaRa) and run on an Mx3005 P sequence detection system, following the instructions of the manufacturer. Primers were designed using Primer 5.0 software (Premier Biosoft International, Palo Alto, CA, USA), and the maize *zSSIIb* gene was employed as the internal control. All primer pairs used for qRT-PCR are listed in Table S6. Data were analyzed using MxPro software.

## Electronic supplementary material

Supporting Information File
