# Supplementary material for: Proteomic analysis of phytase transgenic and non-transgenic maize seeds
Source: Sci Rep. 2017 Aug 23;7:9246. doi: 10.1038/s41598-017-09557-8 (PMC5569035; doi:10.1038/s41598-017-09557-8)
Supplement: Supplementary file 1 — Supporting Information File [file 41598_2017_9557_MOESM1_ESM.pdf]

Proteomic analysis of phytase transgenic and non-transgenic maize seeds

Yanhua Tan[1, 2], Zheng Tong[1], Qian Yang[1], Yong Sun[1], Xiang Jin[1],

Cunzhi Peng[1], Anping Guo[1, 2]*, Xuchu Wang[1, 2]*

1. Key Laboratory of Biology and Genetic Resources for Tropical Crops, Institute of Tropical Biosciences and Biotechnology, Chinese Academy of Tropical Agricultural Sciences, Haikou, Hainan 571101, China
2. College of Agriculture, Hainan University, Haikou, Hainan 570228, China

*Corresponding authors: Dr. Anping Guo and Dr. Xuchu Wang
Chinese Academy of Tropical Agricultural Sciences
Institute of Tropical Biosciences and Biotechnology
Xueyuan Road, Longhua District, Haikou, Hainan 571101, China
Tel.: +86 898 66987460.
E-mail: guoanping@itbb.org.cn; xchwanghainan@163.com

Supporting Information File:

Supplementary Figure S1: The original 2-DE gels for maize seeds.

Supplementary Table S1: MS/MS identification information of DEPs obtained by 2-DE.

Supplementary Table S2: Identification information of DEPs obtained by iTRAQ.

Supplementary Table S3: GO numbers of 148 identified proteins.

Supplementary Table S4: Classification of COG of 148 identified DEPs.

Supplementary Table S5: KEGG pathway enrichment of identified DEPs.

Supplementary Table S6: Primers used in qRT-PCR

Supplementary Figure S1: The typical original 2-DE gels for maize seeds.

NT- Repeat 1

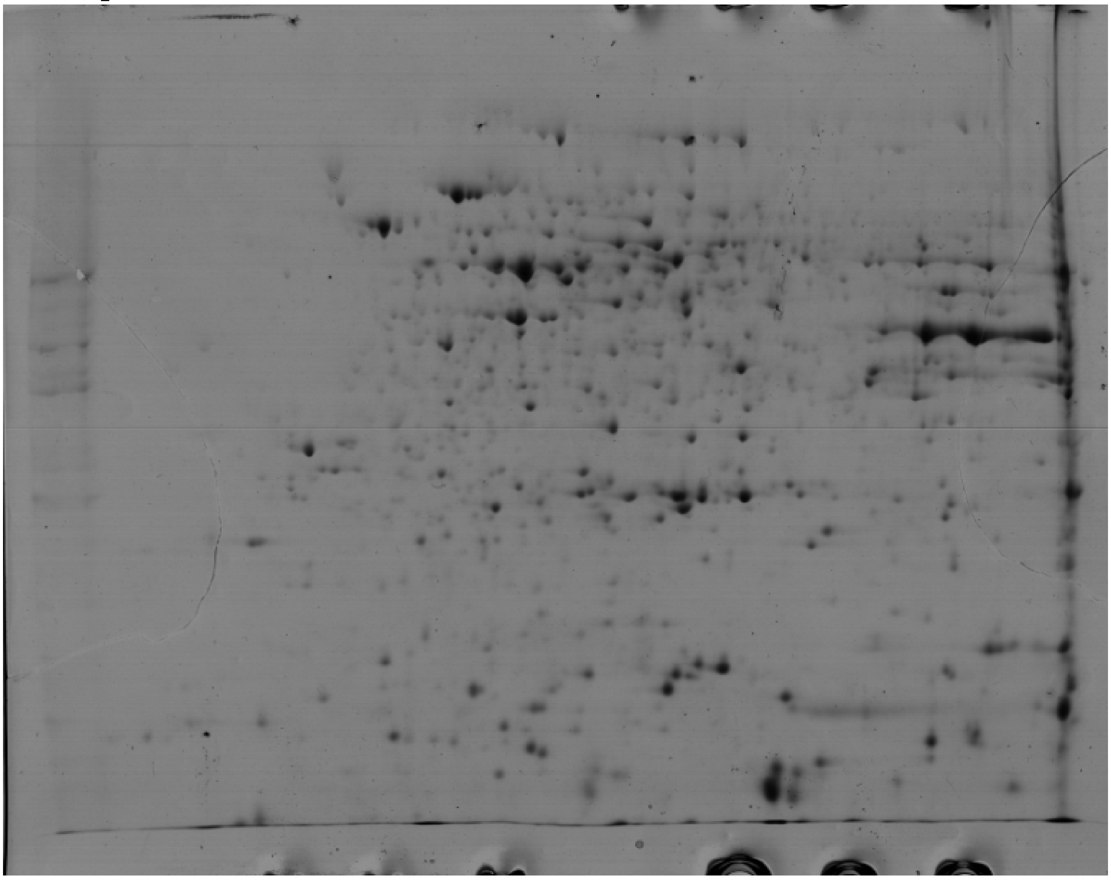

PT-Repeat 1

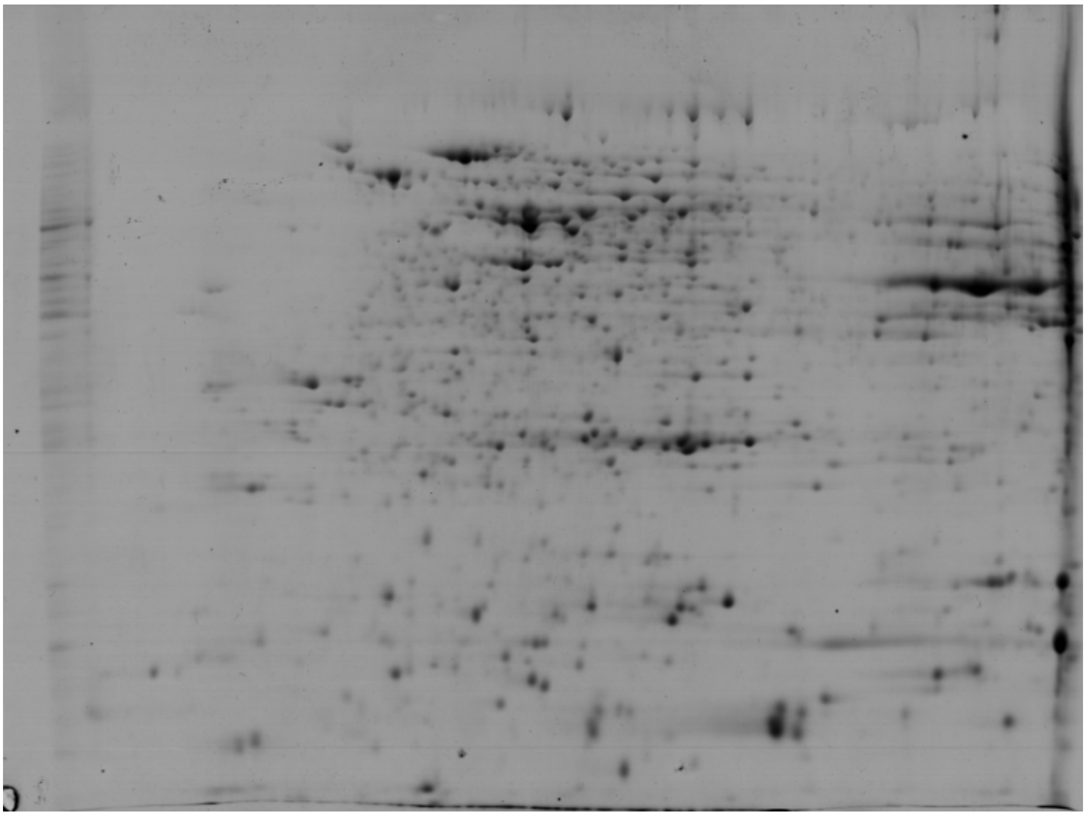

NT- Repeat 2

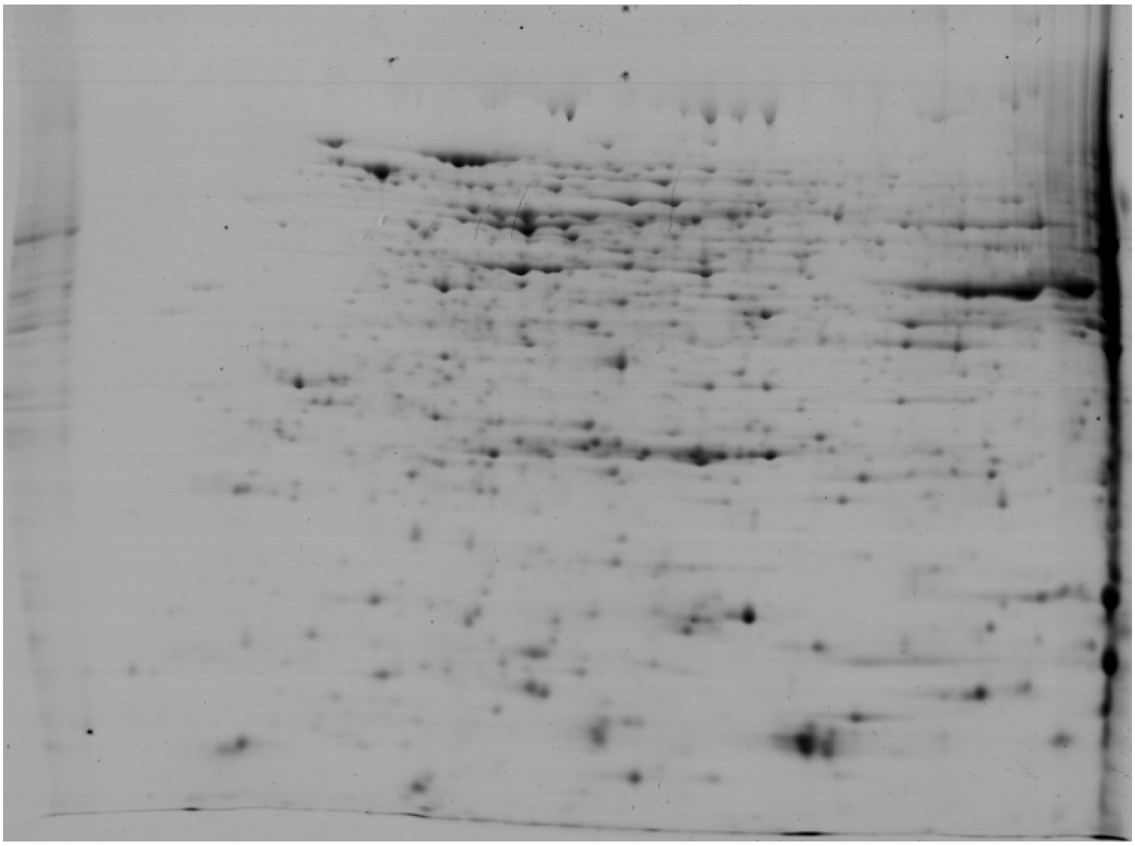

PT- Repeat 2

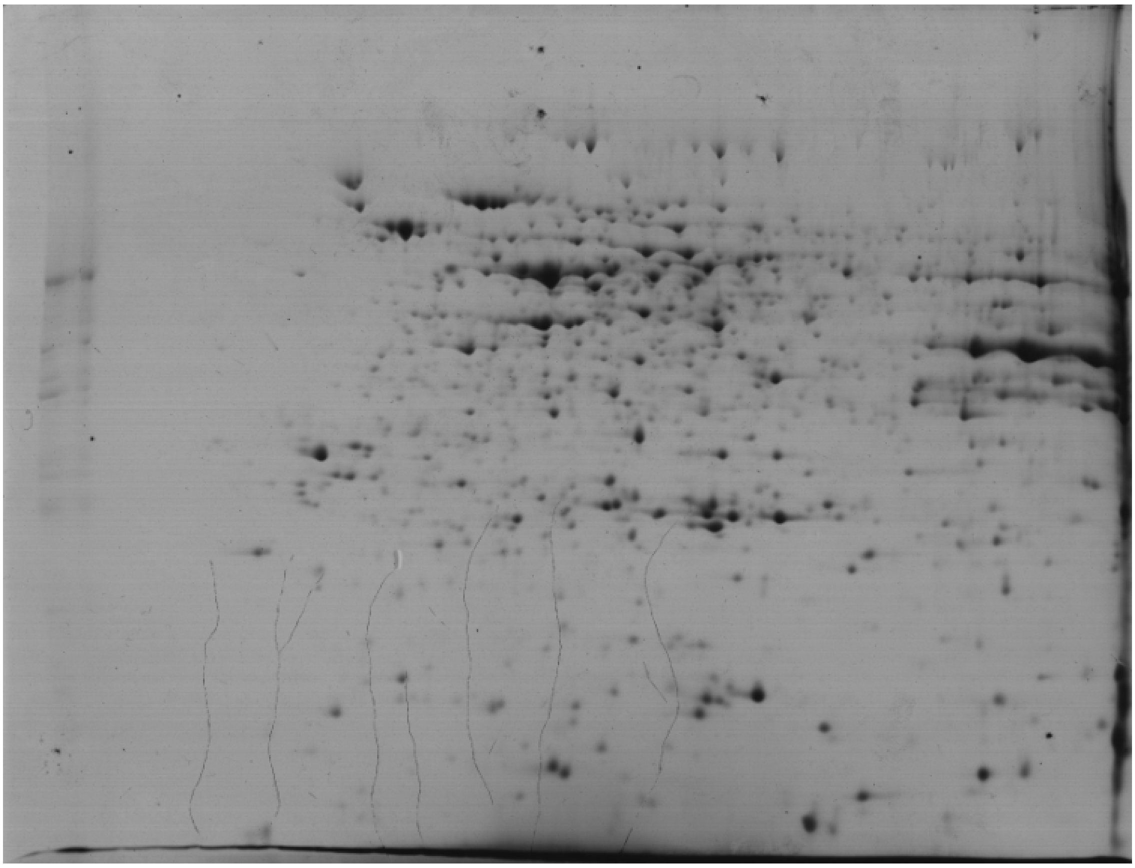

NT- Repeat 3

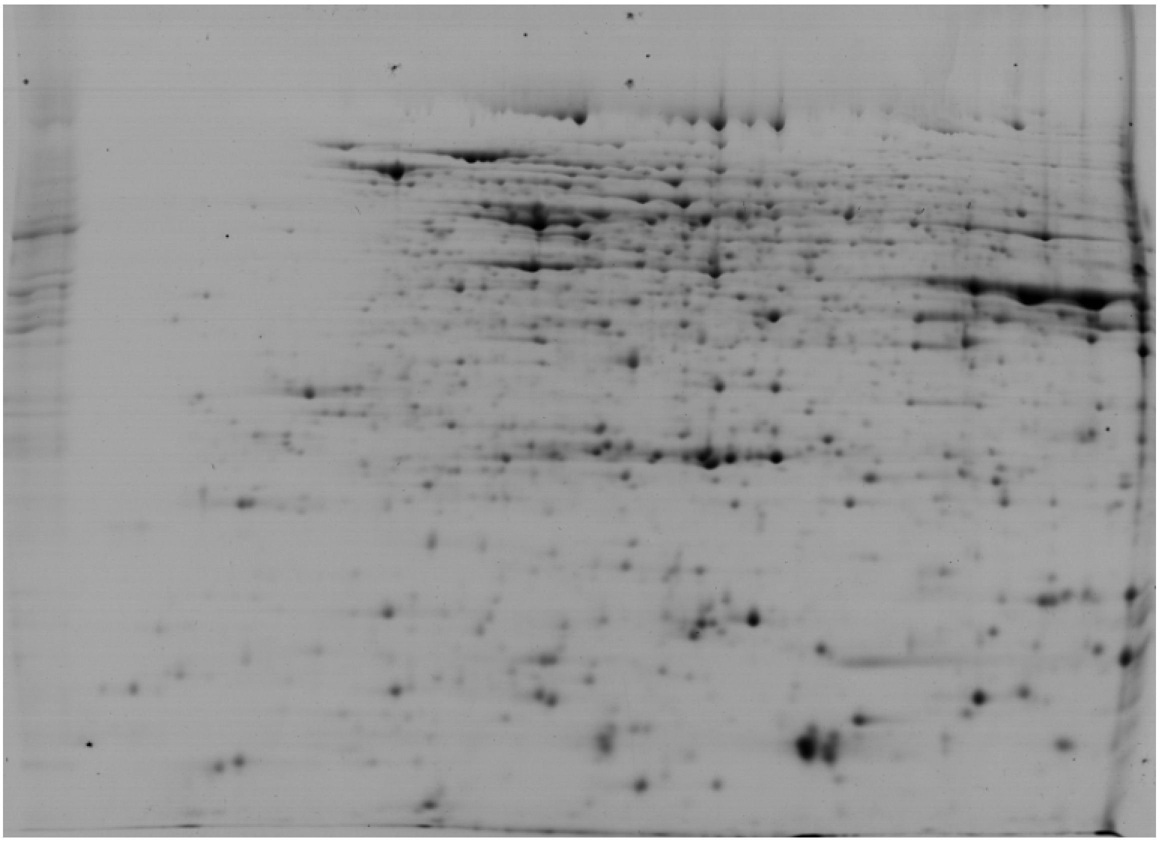

PT- Repeat 3

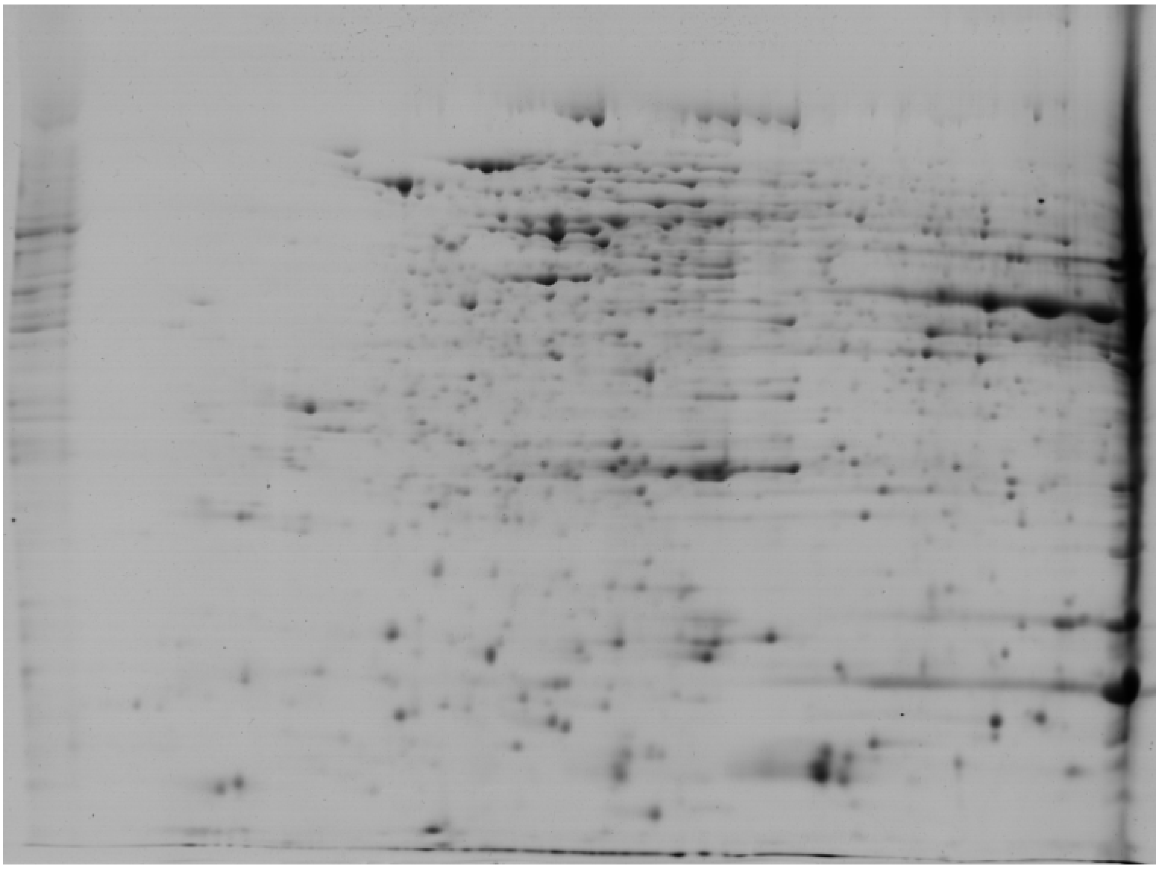

Supplementary Table S1: MS/MS identification information of DEPs obtained by 2-DE.

Spots No	Ac. No	Thero. PI/kDad	MP	Protein Score	Fold change	p value	Protein Description
1	gi|293336560	5.47/60.42	5	591	1.56	1.5E-17	phosphoglycerate mutase1
2	gi|223974403	6.55/50.18	2	102	1.67	0.018	unknown
	gi|226533244	6.55/50.14	2	102		0.018	transaminase/ transferase, transferring nitrogenous groups
3	gi|308080934	5.45/46.82	3	137	1.89	3.0E-04	cytosolic monodehydroascorbate reductase
4	gi|226493582	5.33/38.69	5	198	1.51	8.3E-02	uncharacterized protein LOC100285670
	gi|226508732	5.38//39.01	3	124		0.075	activator of 90 kDa heat shock protein ATPase
	gi|413921986	5.95/49.32	3	124		0.083	activator of heat shock protein ATPase
5	gi|413918083	4.83/39.35	3	214	1.81	2.E-08	eukaryotic translation initiation factor 3 subunit 3
	gi|226493027	4.83/39.42	3	214		2.E-08	uncharacterized protein LOC100280503
	gi|413918085	4.83/38.59	3	214		2.E-08	hypothetical protein ZEAMMB73_519156
	gi|670442963	4.83/39.23	3	214		2.E-08	PREDICTED: LOC100280503 isoform X1
6	gi|162461501	6.41/36.63	5	520	2.04	5.5E-10	glyceraldehyde-3-phosphate dehydrogenase 2, cytosolic
	gi|194700850	6.40/32.08	5	520		5.5E-10	unknown
	gi|413941770	6.67/37.27	5	520		5.5E-10	glyceraldehyde-3-phosphate dehydrogenase2
	gi|413921389	6.45/32.03	3	240		1.20E-08	glyceraldehyde-3-phosphate dehydrogenase1 isoform 1
7	gi|413916077	6.89/21.80	3	278	1.94	9.0E-11	hypothetical protein ZEAMMB73_718489
	gi|308080598	5.86/25.67	3	278		9.0E-11	uncharacterized protein LOC100502283
8	gi|226532150	5.04/26.68	2	90	1.73	0.05	isoamyl acetate-hydrolyzing esterase
9	gi|195605636	5.53/27.28	2	157	1.57	8.90E-06	triosephosphate isomerase, cytosolic
	gi|670440305	6.31/71.39	3	382		5.00E-11	PREDICTED: globulin-1 S allele-like
10	gi|226498252	5.14/19.56	4	392	1.55	1.6E-11	adenine phosphoribosyltransferase 1
	gi|414877870	8.93/24.14	4	392		1.6E-11	TPA: adenine phosphoribosyltransferase 1
	gi|194701624	5.14/19.51	4	392		1.6E-11	unknown
11	gi|413944599	5.38/18.20	3	180	1.61	1.3E-05	USP family protein
	gi|212721114	5.63/18.30	3	180		1.3E-05	uncharacterized protein LOC100194018
12	gi|413949326	6.04/103.78	4	84	0.66	0.34	pyruvate, phosphate dikinase 1, Precursor
13	gi|413949326	6.04/103.78	11	487	0.53	2.2E-05	pyruvate, phosphate dikinase 1, Precursor
14	gi|413949326	5.81/64.69	2	198	0.53	1.0E-09	ruBisCO large subunit-binding protein subunit beta
	gi|226493641	5.81/64.52	2	198		1.0E-09	ruBisCO large subunit-binding protein subunit beta
	gi|413926905	5.81/64.62	2	198		1.0E-09	putative TCP-1/cpn60 chaperonin family protein isoform 1
15	gi|189027076	6.16/57.89	7	740	0.52	1.9E-12	glucose-1-phosphate adenylyltransferase large subunit 1
16	gi|217330650	5.29/42.16	3	233	0.66	7.2E-08	actin related protein 1
17	gi|169245755	5.25/39.57	2	186	0.64	9.1E-09	glutamine synthetase
	gi|162463755	5.34/39.57	2	186		9.1E-09	glutamine synthetase root isozyme 3
18	gi|194703858	5.50/39.20	4	157	0.59	0.0035	gibberellin 20 oxidase 2

19	gi|414586767	5.46/19.71	2	157	0.54	7.5E-06	TPA: hypothetical protein ZEAMMB73_925834
	gi|162460084	5.25/34.66	2	157		7.5E-06	L-galactose dehydrogenase
20	gi| 226508112	6.97/41.84	3	194	0.66	8.3E-08	cysteine synthase1
21	gi|219887709	6.77/40.13	5	293	0.62	2.9E-06	unknown
22	gi|195650645	6.16/32.83	4	401	0.49	6.7E-10	carbonyl reductase 1
23	gi|224029461	5.08/28.96	2	184	0.66	4.9E-09	6-phosphogluconolactonase
24	gi|226504576	5.65/27.48	3	173	0.62	1.6E-04	APx1 - Cytosolic Ascorbate Peroxidase
25	gi|224029787	6.10/27.51	4	311	0.47	1.1E-07	proteasome subunit alpha type
26	gi|194704678	5.95/21.60	4	295	0.60	6.2E-07	minor allergen Alt a 7
27	gi|4468794	5.96/23.87	5	408	0.53	2.2E-13	glutathione transferase III(b)
28	gi|195605616	4.53/18.79	5	327	0.54	1.9E-07	translationally-controlled tumor protein
29	gi|195605616	4.54/18.79	3	182	0.33	2.5E-04	translationally-controlled tumor protein
30	gi|226504982	8.34/21.74	2	110	0.66	5.5E-04	eukaryotic translation initiation factor 1A
31	gi|840086919	6.30/16.53	2	153	0.65	1.0E-07	nucleoside diphosphate kinase 1
32	gi|266398	8.07/16.86	3	181	0.47	3.0E-04	trypsin/factor XIIA inhibitor

Note: 1) Spots No is assigned spot numbers as indicated in Figure 2.

2) Best Protein Accession is according to UNIProt.

3)Thero.kDad and Thero. pI are the theoretical values of molecular weight (Mr., kDa) and *p*I for the identified proteins.

4) MP is the numbers of the matched peptides

5) Percent values of coverage (%) of the matched peptides in the whole protein sequence.

6) p value is the highest score's Expect of the matched peptides.

7) Fold change is the ratio of PT maize seeeds compared to NT maize seeds.

Supplementary Table S2: Identification information of DEPs obtained by iTRAQ.

Protein No.	Unused Score	Cov. (95%)	Peptides (95%)	Fold (PT/NT)	p value	Accession	Name
33	3.09	44.64	5	0.0450001	0.04354	tr|A3KLI1|A3KLI1_MAIZE	RAB17 protein
34	16	68.13	16	0.0672377	0.04012	tr|B6SI37|B6SI37_MAIZE	Embryonic abundant protein 1
35	74.43	51.92	81	0.0942339	0.01667	tr|C0PGM3|C0PGM3_MAIZE	Uncharacterized protein
36	32.89	57.5	25	0.1023999	0.01005	tr|Q41815|Q41815_MAIZE	Heat shock protein 26
37	61.65	60.44	118	0.11171	0.01099	tr|Q7M1Z8|Q7M1Z8_MAIZE	Globulin-2
38	30.63	61.66	243	0.1452166	0.0023	tr|K7VJF3|K7VJF3_MAIZE	Uncharacterized protein
39	10.11	29.07	6	0.1524359	0.00673	tr|B8QY31|B8QY31_ZEAMP	Zeamatin-like protein
40	17.79	18.83	12	0.190091	0.00417	tr|B6SLF6|B6SLF6_MAIZE	Antimicrobial peptide MBP-1
41	6.17	40.74	12	0.2084298	0.03346	tr|B6TLK8|B6TLK8_MAIZE	17.4 kDa class I heat shock protein 3
42	12	62.83	9	0.2109165	0.02405	tr|B6T8E4|B6T8E4_MAIZE	Embryonic abundant protein 1
43	7.11	26.71	4	0.2141356	0.00021	tr|B6SQM6|B6SQM6_MAIZE	Pathogeneis protein 10
44	46.84	38.57	57	0.2189845	0.02633	tr|K7W272|K7W272_MAIZE	Uncharacterized protein
45	3.08	84.6	133	0.2194325	0.02721	tr|Q4U168|Q4U168_MAIZE	Glucose-1-phosphate adenylyltransferase
46	20.01	21.69	12	0.2400357	0.0391	tr|A0A096TN72|A0A096TN72_MAIZE	Uncharacterized protein
47	17.38	30.03	14	0.2451414	0.0326	tr|B4FKP4|B4FKP4_MAIZE	Uncharacterized protein
48	75.08	59.21	88	0.2507435	0.02544	tr|Q9S822|Q9S822_MAIZE	Heat shock protein 101
49	17.66	35.71	9	0.2664746	0.01618	tr|B6UGU4|B6UGU4_MAIZE	Steroleosin
50	7.08	38.98	5	0.3019117	0.00468	tr|B6UGS5|B6UGS5_MAIZE	Putative uncharacterized protein
51	22.07	50.42	22	0.3056299	0.01059	tr|B4FFZ9|B4FFZ9_MAIZE	Lipoprotein
52	33.25	46.81	19	0.3181477	0.02409	tr|Q8S532|Q8S532_MAIZE	Cytosolic aldehyde dehydrogenase

53	10.02	35.43	11	0.3182029	0.03493	tr|C0P5U8|C0P5U8_MAIZE	Glutathione S-transferase 4
54	36.75	45.21	29	0.324057	0.00384	tr|B6SK46|B6SK46_MAIZE	Cupin family protein
55	18.9	47.33	23	0.3250873	0.01758	tr|C0P451|C0P451_MAIZE	Endochitinase B
56	10.22	41.89	35	0.3299068	0.01038	tr|B6TSQ9|B6TSQ9_MAIZE	Aspartic proteinase oryzasin-1
57	0.12	2.448	2	0.3308785	0.03503	tr|B6SSB8|B6SSB8_MAIZE	Transposon protein
58	8	49.21	4	0.3314464	0.04201	tr|K7WCU4|K7WCU4_MAIZE	Uncharacterized protein
59	28.25	78.17	29	0.3346054	0.0204	tr|B4FFK9|B4FFK9_MAIZE	Uncharacterized protein
60	12.19	59.09	27	0.3416796	0.04213	tr|C0PPC9|C0PPC9_MAIZE	Uncharacterized protein
61	26.79	65.58	21	0.3450231	0.02513	tr|Q19VG6|Q19VG6_MAIZE	Major latex protein 22
62	4.91	7.616	4	0.3453195	0.03303	tr|A0A096RYL4|A0A096RYL4_MAIZE	Uncharacterized protein
63	7.53	15.96	5	0.3515269	0.01356	tr|K7V5N4|K7V5N4_MAIZE	Uncharacterized protein
64	2.7	60.24	65	0.3529589	0.0499	tr|E9NQE3|E9NQE3_MAIZE	Malate dehydrogenase
65	10.58	39.2	22	0.3612606	0.02839	tr|B6UGN9|B6UGN9_MAIZE	Oleosin
66	40.12	59.56	24	0.3636518	0.01022	tr|B4F9K0|B4F9K0_MAIZE	Uncharacterized protein
67	6.55	21.17	9	0.3646264	0.03897	tr|B8A3K0|B8A3K0_MAIZE	Uncharacterized protein
68	13.44	73.24	11	0.3731083	0.03943	tr|B4FJM6|B4FJM6_MAIZE	Uncharacterized protein
69	8.26	70	9	0.3737725	0.01297	tr|A0A096RTN1|A0A096RTN1_MAIZE	Uncharacterized protein
70	13.72	42.24	9	0.379674	0.04938	tr|B6U9I3|B6U9I3_MAIZE	Eukaryotic translation initiation factor 3
71	26.06	52.88	19	0.3979553	0.03314	tr|B4G1C2|B4G1C2_MAIZE	Uncharacterized protein
72	6.01	15.74	5	0.3999321	0.00782	tr|B6SGN7|B6SGN7_MAIZE	Embryonic protein
73	14.84	47.13	11	0.4169755	0.00545	tr|B6TTP4|B6TTP4_MAIZE	Stress-inducible membrane pore protein
74	23.87	39.18	16	0.4224408	0.02245	tr|K7UTW6|K7UTW6_MAIZE	Plasminogen activator inhibitor

75	6	22.88	4	0.4304308	0.03014	tr|B6UDH8|B6UDH8_MAIZE	Putative uncharacterized protein
76	50.92	54.88	65	0.4327371	0.02182	tr|Q8S4W8|Q8S4W8_MAIZE	Pyruvate decarboxylase
77	48.24	59.84	44	0.4445181	0.02871	tr|B4FAL8|B4FAL8_MAIZE	Uncharacterized protein
78	6.38	12.53	4	0.4612433	0.01903	tr|B6TII3|B6TII3_MAIZE	ADP-ribosylation factor GTPase-activating protein 3
79	6	26.32	6	0.4617479	0.01633	tr|B6TKN6|B6TKN6_MAIZE	Nonspecific lipid-transfer protein
80	15.89	53.17	34	0.466296	0.03661	tr|B6TF92|B6TF92_MAIZE	Stress responsive protein
81	15.33	23.06	10	0.4666924	0.0329	tr|C0HFV7|C0HFV7_MAIZE	Putative apyrase family protein
82	1.87	4.021	3	0.4763553	0.02505	tr|B8A0H4|B8A0H4_MAIZE	Uncharacterized protein
83	10.01	36.84	9	0.4830587	0.04199	tr|B4F9E8|B4F9E8_MAIZE	Uncharacterized protein
84	0.18	17.13	18	0.4924985	0.01502	tr|A0A096SBR2|A0A096SBR2_MAIZE	Uncharacterized protein
85	1.02	42.53	4	0.5001847	0.02501	tr|K7TQT5|K7TQT5_MAIZE	Uncharacterized protein
86	7.14	10.76	7	0.5063184	0.00556	tr|K7V5M7|K7V5M7_MAIZE	Uncharacterized protein
87	2	7.659	2	0.5086174	0.00244	tr|A0A096RDK8|A0A096RDK8_MAIZE	Uncharacterized protein
88	41.5	78.22	30	0.5197755	0.03317	tr|C0HFQ1|C0HFQ1_MAIZE	Uncharacterized protein
89	14.24	25.87	9	0.5199078	0.02441	tr|A0A096TAP2|A0A096TAP2_MAIZE	Uncharacterized protein
90	87.32	48.67	69	0.5247852	0.01234	tr|K7VD78|K7VD78_MAIZE	Putative chaperone clbp family protein
91	10.67	24.93	7	0.5277387	0.01411	tr|B6UHC9|B6UHC9_MAIZE	Putative uncharacterized protein
92	18.89	42.91	11	0.544157	0.0376	tr|B4FRD1|B4FRD1_MAIZE	Adaptin ear-binding coat-associated protein 1
93	19.45	46.82	13	0.5519891	0.02685	tr|B6THJ5|B6THJ5_MAIZE	Phosphosulfolactate synthase-related protein
94	4.61	18.31	5	0.5532653	0.00692	tr|B6UGM9|B6UGM9_MAIZE	Putative uncharacterized protein
95	5.89	12.61	7	0.5535207	0.01222	tr|A0A096TH62|A0A096TH62_MAIZE	Uncharacterized protein
96	5.82	23.1	8	0.5556986	0.0415	tr|C0HI30|C0HI30_MAIZE	Uncharacterized protein

97	4.05	3.289	3	0.5583078	0.03175	tr|A0A096QKY4|A0A096QKY4_MAIZE	Uncharacterized protein
98	2.06	28.02	12	0.5617748	0.0022	tr|B4FL64|B4FL64_MAIZE	Ribosomal protein L19
99	81.97	55.44	61	0.564937	0.02928	tr|Q9AXG8|Q9AXG8_MAIZE	Lipoxygenase
100	4.09	7.818	2	0.5683276	0.00435	tr|B6TRW7|B6TRW7_MAIZE	Sigma factor sigB regulation protein rsbQ
101	11.01	21.19	9	0.5782984	0.00033	tr|B4G0U5|B4G0U5_MAIZE	Uncharacterized protein
102	3.18	9.502	4	0.5802784	0.01508	tr|B6TA85|B6TA85_MAIZE	Protein binding protein
103	25.61	25.79	20	0.5904589	0.01453	tr|A0A096SXV7|A0A096SXV7_MAIZE	Uncharacterized protein
104	0.29	2.361	2	0.5941475	0.03937	tr|A0A096PME5|A0A096PME5_MAIZE	Uncharacterized protein
105	9.97	25.37	6	0.5954451	0.04405	tr|B7ZZM7|B7ZZM7_MAIZE	Uncharacterized protein
106	37.55	34.18	25	0.5975416	0.00607	tr|A0A096QF11|A0A096QF11_MAIZE	Uncharacterized protein
107	14.11	9.063	8	0.6060148	0.00095	tr|A0A096TMH0|A0A096TMH0_MAIZE	Uncharacterized protein
108	12.26	13.43	13	0.6110004	0.00712	tr|A0A096QBA0|A0A096QBA0_MAIZE	Uncharacterized protein
109	22.26	44.65	16	0.6119334	0.02979	tr|C0PF66|C0PF66_MAIZE	Aminoacylase-1
110	23.53	36.49	14	0.6225439	0.04776	tr|B4FEU5|B4FEU5_MAIZE	Uncharacterized protein
111	17.28	57.35	13	0.6251728	0.00879	tr|B6U8P6|B6U8P6_MAIZE	ABA-responsive protein
112	2.04	24.45	7	0.6357828	0.04034	tr|B4FSU1|B4FSU1_MAIZE	Transmembrane emp24 domain-containing protein 10
113	39.54	39.72	21	0.6366875	0.03225	tr|Q5G1U0|Q5G1U0_MAIZE	Malic enzyme
114	87.46	54.14	87	0.6392265	0.03561	tr|B6SV64|B6SV64_MAIZE	Heat shock 70 kDa protein 4
115	21.81	32.3	24	0.6448073	0.03566	tr|B4G1G1|B4G1G1_MAIZE	Desiccation-related protein
116	12.98	26.63	7	0.6457979	0.02395	tr|B6SMK8|B6SMK8_MAIZE	Inositol-1-monophosphatase
117	4.02	7.2	2	0.6560965	0.01143	tr|A0A096THV6|A0A096THV6_MAIZE	Uncharacterized protein
118	17.23	48.22	11	1.518215	0.00699	tr|B6T346|B6T346_MAIZE	THO complex subunit 4

119	13.43	14.39	9	1.5254947	0.03281	tr|K7TNE7|K7TNE7_MAIZE	Uncharacterized protein
120	16.15	48.24	12	1.534568	0.01938	tr|B4G1B0|B4G1B0_MAIZE	Remorin
121	2.04	8.285	4	1.5482753	0.04145	tr|Q32SG1|Q32SG1_MAIZE	Protein phosphatase 2A regulatory subunit B
122	2	35.19	23	1.5533969	0.00237	tr|B4FBP3|B4FBP3_MAIZE	Signal recognition particle subunit SRP68
123	31.92	48.31	21	1.561641	0.02769	tr|B4FU39|B4FU39_MAIZE	Uncharacterized protein
124	2	10.64	4	1.5679684	0.02655	tr|K7UYP8|K7UYP8_MAIZE	Basic endochitinase A
125	2.29	20.33	4	1.5683207	0.02247	tr|B6SX73|B6SX73_MAIZE	60S ribosomal protein L35
126	26.98	64.46	15	1.5760592	0.02578	tr|B4F957|B4F957_MAIZE	Uncharacterized protein
127	52.67	28.18	35	1.5827443	0.02844	tr|K7U005|K7U005_MAIZE	Uncharacterized protein
128	18.95	42.58	18	1.6150435	0.03952	tr|B6UHJ9|B6UHJ9_MAIZE	60S ribosomal protein L23a
129	25.26	42.58	38	1.6415858	0.03679	tr|B4FSQ4|B4FSQ4_MAIZE	40S ribosomal protein SA
130	1.31	29.25	8	1.6464081	0.00403	tr|B6TA79|B6TA79_MAIZE	Cell division control protein 2
131	38.98	50.78	34	1.6957221	0.03759	tr|A0A096QRB1|A0A096QRB1_MAIZE	Uncharacterized protein
132	27.05	69.49	271	1.6958159	0.02905	tr|B7ZZ42|B7ZZ42_MAIZE	Uncharacterized protein
133	60.92	56.5	91	1.7201191	0.01306	tr|Q6R987|Q6R987_MAIZE	ATP synthase subunit alpha
134	14.15	70.27	8	1.7296883	0.03688	tr|B6SMY5|B6SMY5_MAIZE	Putative uncharacterized protein
135	30.84	60.98	27	1.7584882	0.03362	tr|B4F848|B4F848_MAIZE	Chaperonin
136	37.4	37.79	21	1.7693588	0.04681	tr|K7U1M0|K7U1M0_MAIZE	Uncharacterized protein
137	3.25	9.626	2	1.7778263	0.04074	tr|B6T6D7|B6T6D7_MAIZE	MIR-interacting saposin-like protein
138	10.02	57.69	9	1.7782231	0.01423	tr|B6SIF0|B6SIF0_MAIZE	Glycine-rich RNA-binding protein 2
139	17.09	37.04	11	1.9064306	0.03986	tr|B6T9E1|B6T9E1_MAIZE	Xyloglucan endotransglucosylase/hydrolase
140	13.97	48.44	8	1.93203	0.00901	tr|B4FTE1|B4FTE1_MAIZE	Signal peptidase complex subunit 2

141	12.11	11.51	9	1.946139	0.000 98	tr|A0A096QQ54|A0A09 6QQ54_MAIZE	Uncharacterized protein
142	5.11	25.98	5	1.972951 8	0.030 21	tr|B6SIP0|B6SIP0_MAI ZE	NHP2-like protein 1
143	20.56	39.83	15	1.976405 7	0.049 14	tr|P93852|P93852_MAI ZE	Cycloartenol-C-24-methyltransferase 1
144	14.54	32.37	9	1.991969	0.011 63	tr|A0A096RS97|A0A09 6RS97_MAIZE	Uncharacterized protein
145	14.04	33.21	8	2.033743 6	0.001 86	tr|K7VTU7|K7VTU7_M AIZE	Uncharacterized protein
146	8.6	45.35	18	2.088233	0.013 22	tr|Q9ZQX9|Q9ZQX9_M AIZE	40S ribosomal protein S27
147	19.26	32.78	15	4.092953 7	0.012 58	tr|B6UD26|B6UD26_M AIZE	O-methyltransferase ZRP4
148	10	18.22	6	9.260415 9	0.033 25	tr|C4J5L2|C4J5L2_MAI ZE	Uncharacterized protein

Note:

1) Protein Number is followed the spot numbers as showed in Table S1.

2) Best Protein Accession is according to UNIProt.

3) MP is the numbers of the matched peptides

4) Percent values of coverage (%) of the matched peptides in the whole protein sequence.

Supplementary Table S3: GO numbers of the 148 identified proteins.

Q9AXG8	GO:0050832	GO:0002215	GO:0046872	GO:0016702	GO:0031408	
B6T9E1	GO:0048046	GO:0005618	GO:0042546	GO:0071555	GO:0004553	GO:0010411
A0A096SXV7	GO:0004559	GO:0030246	GO:0006013	GO:0008270		
Q6R987	GO:0005524	GO:0015991	GO:0015986	GO:0005739	GO:0046933	GO:0045261
Q5G1U0	GO:0051287	GO:0004471	GO:0006108	GO:0046872		
P93852	GO:0006084	GO:0016132	GO:0042398	GO:0009805	GO:0009793	GO:0005783
C0PF66	GO:0004046	GO:0006520	GO:0005737	GO:0046872	GO:0008237	
Q9ZTQ4	GO:0005975	GO:0004575				
A0A096TAP2	GO:0004185					
E9NQE3	GO:0030060	GO:0005975	GO:0006108	GO:0006099		
B4FSQ4	GO:0022627	GO:0000028	GO:0003735	GO:0006412		
Q9S822	GO:0005524	GO:0019538				
B6U9I3	GO:0016282	GO:0033290	GO:0005852	GO:0001731	GO:0006446	GO:0003743
B4FBP3	GO:0008312	GO:0006614	GO:0030942	GO:0005047	GO:0005786	
B6UGN9	GO:0016021	GO:0012511				
Q9ZQX9	GO:0046872	GO:0005840	GO:0003735	GO:0006412		
Q32SG1	GO:0000159	GO:0008601	GO:0007165			
A0A096QF11	GO:0009738	GO:0005622	GO:0035556	GO:0019243	GO:0010583	GO:0009723
B6TSQ9	GO:0004190	GO:0006629				
B4FSU1	GO:0016021	GO:0006810				
K7VD78	GO:0005524	GO:0005737	GO:0016485	GO:0009408		
B4FL64	GO:0005840					

		GO:0003735	GO:0006412			
B6THJ5	GO:0003824	GO:0019295	GO:0010286	GO:0010608	GO:0006457	GO:0009644
A0A096RS97	GO:0046872	GO:0016491				
B6SSB8	GO:0020037	GO:0005506	GO:0004497	GO:0016705		
K7TNE7	GO:0000911	GO:0000226	GO:0005643	GO:0006611	GO:0009627	
P15590	GO:0045735					
P31927	GO:0005986	GO:0016157	GO:0046524			
M7ZGS7	GO:0016811					
Q94J20						
B6SIP0	GO:0003723	GO:0030529	GO:0005730	GO:0042254		
B6SX73	GO:0005840	GO:0003735	GO:0006412			
B4F922	GO:0010143	GO:0051179	GO:0016747	GO:0010090		
Q19VG6	GO:0006952	GO:0009607				
K7W272	GO:0045735					
B6SQM6	GO:0006952	GO:0009607				
B8A0H4						
B4FU39	GO:0016491	GO:0008270				
B4FRD1	GO:0006897	GO:0016020				
B4F848	GO:0005524	GO:0005737	GO:0006457			
B6TA85						
B4F957						
B6T6D7	GO:0034976	GO:0009627				
B6TA79	GO:0005524	GO:0051301	GO:0004672			
B6SLF6	GO:0050832					
B6TII3	GO:0005096					
B6T8E4						

B6SGN7					
A3KLI1	GO:0006950	GO:0009415			
B4G1C2	GO:0005975	GO:0004553			
B4FGK8	GO:0005850	GO:0003743			
K7UTW6					
C0P5U8	GO:0016740				
B6UHJ9	GO:0000166	GO:0005840	GO:0003735	GO:0006412	
Q41815	GO:0010286	GO:0006457			
B6TTP4	GO:0016021				
K7UYP8	GO:0005975	GO:0016998	GO:0008061	GO:0006032	GO:0004568
B8A3K0					
K7V5M7					
C0HFV7	GO:0016787				
B4G1G1					
B4FTE1	GO:0016021	GO:0008233	GO:0005787	GO:0006465	
B6SIF0	GO:0003676	GO:0000166			
C0HFQ1					
B6SMK8	GO:0004401	GO:0046854			
K7V5N4	GO:0004190				
Q7M1Z8	GO:0045735				
B6T346	GO:0003676	GO:0000166			
A0A096QKY4					
Q8S4W8	GO:0016831	GO:0000287	GO:0030976		
B4F9E8					
K7VTU7	GO:0005576	GO:0019953			
C0P451	GO:0005975	GO:0016998	GO:0008061	GO:0006032	GO:0004568
B6TRW7	GO:0010223				

		GO:1901601		
B4G1B0				
B6SI37				
Q8S532	GO:0016620			
K7WCU4				
K7U1M0	GO:0003885	GO:0050660	GO:0016020	
B6TF92				
B6SJE9				
B6UGU4	GO:0016491			
M8CBN1	GO:0007010	GO:0005829	GO:0006094	GO:0010498
A0A059PZ24	GO:0016021			
B9RET0	GO:0030127	GO:0006888	GO:0006886	GO:0008270
B6TFC2	GO:0008430			
B6UDH8				
A0A096RTN1	GO:0006952	GO:0009607		
B6SV64	GO:0005524			
A0A096QRB1	GO:0005524	GO:0005737	GO:0016787	GO:0030163
B6STY9	GO:0009269			
B6TLK8				
B6T871	GO:0016829			
B6UGS5	GO:0006950	GO:0009415		
A0A096QQ54	GO:0046872	GO:0004222		
C4J004	GO:0010223	GO:1901601		
B6TXL1				
B6TKN6				
Q7XZQ2	GO:0003978	GO:0006012		
A0A096TN72				
B6SMY5				
B6UHC9	GO:0016491			

B6U8P6						
Q41886	GO:0045735					
B6SK46	GO:0045735					
C4J5L2	GO:0016758					
B6TXJ8	GO:0005975	GO:0071555	GO:0005576	GO:0004650		
A0A096PME5						
B6UD26	GO:0008171					
A0A096TH62						
Q4U168	GO:0008878	GO:0005978				
A0A0A7LS12						
X4Z319	GO:0005524					
B8QY31						
C6F1K2	GO:0005524					
Q10EF5						
B8A306	GO:0005737	GO:0006007	GO:0030145	GO:0004619		
B6TIG6	GO:0010285	GO:0009089	GO:0030170			
Q9XFZ3	GO:0050660	GO:0016656				
B4FNW7	GO:0001671	GO:0006950				
P01088	GO:0005576	GO:0004867				
B4FS87	GO:0051287	GO:0050661	GO:0006006	GO:0016620		
C4J9Y2						
B6THH7						
B6SIG5	GO:0006096	GO:0004807				
B6T920	GO:0003999	GO:0006168	GO:0005737	GO:0009116		
A0A096TB53	GO:0006950					
P55241	GO:0005524	GO:0009501	GO:0009507	GO:0008878	GO:0005978	GO:0019252
Q9XFZ3	GO:0050660	GO:0016656				
B6THN2	GO:0005524	GO:0005737	GO:0042026			
K7TNP5	GO:0016282	GO:0033290	GO:0005852	GO:0001731	GO:0003743	
X1X262	GO:0005524					
B9TSW1	GO:0005524	GO:0004356	GO:0006542			
B6TF99	GO:0005506	GO:0016491				
Q1PBI2						

B8A367	GO:0006535	GO:0004124	GO:0016740		
B8A230					
B6U607					
B6UAK0	GO:0017057	GO:0005975	GO:0006098		
B6UB73	GO:0020037	GO:0004601	GO:0006979		
C0PFK3	GO:0005737	GO:0005634	GO:0019773	GO:0004298	GO:0006511
B4FWD0	GO:0010181	GO:0045892	GO:0016491		
Q9SM20	GO:0004364				
B6SIF5					
B6SIF5					
B6T9H2	GO:0016021	GO:0003743			
B4FK49	GO:0005524	GO:0006241	GO:0006183	GO:0006228	GO:0004550
K7VVI6	GO:0005524	GO:0016301	GO:0046872	GO:0006090	GO:0050242

Supplementary Table S4: Classification of COG of 148 identified DEPs.

Protein No	Accession	Protein Name	Species
**RNA processing and modification(A)**			
86	B6T346_MAIZE	THO complex subunit 4	MAIZE
110	B6SIP0_MAIZE	NHP2-like protein 1	MAIZE
**Energy production and conversion(C)**			
20	Q8S532_MAIZE	Cytosolic aldehyde dehydrogenase RF2C	MAIZE
32	E9NQE3_MAIZE	Malate dehydrogenase	MAIZE
53	B6TXL1_MAIZE	Mitochondrial NADH ubiquinone oxidoreductase 13kD-like subunit	MAIZE
55	M7ZGS7_TRIUA	Formamidase	Triticum urartu
81	Q5G1U0_MAIZE	Malic enzyme	MAIZE
101	Q6R987_MAIZE	ATP synthase subunit alpha	MAIZE
104	K7U1M0_MAIZE	L-gulonolactone oxidase	MAIZE
116	C4J5L2_MAIZE	Glycosyltransferase	MAIZE
128	K7VVI6_MAIZE	pyruvate, phosphate dikinase 1, Precursor	MAIZE
129	K7VVI6_MAIZE	pyruvate, phosphate dikinase 1, Precursor	MAIZE
135	Q1PBI2_MAIZE	L-galactose dehydrogenase	MAIZE
140	B6UB73_MAIZE	APx1 - Cytosolic Ascorbate Peroxidase	MAIZE
**Amino acid transport and metabolism(E)**			
44	Q8S4W8_MAIZE	Pyruvate decarboxylase	MAIZE
67	Q9AXG8_MAIZE	Lipoxygenase	MAIZE
77	C0PF66_MAIZE	Aminoacylase-1	MAIZE
118	B6TIG6_MAIZE	transaminase/ transferase, transferring nitrogenous groups	MAIZE
133	B9TSW1_MAIZE	glutamine synthetase	MAIZE
136	B8A367_MAIZE	cysteine synthase1	MAIZE
**Nucleotide transport and metabolism(F)**			
49	C0HFV7_MAIZE	Putative apyrase family protein	MAIZE
126	B6T920_MAIZE	adenine phosphoribosyltransferase 1	MAIZE
147	B4FK49_MAIZE	nucleoside diphosphate kinase 1	MAIZE
**Carbohydrate transport and metabolism(G)**			
5	Q7M1Z8_MAIZE	Globulin-2	MAIZE
23	C0P451_MAIZE	Endochitinase B	MAIZE
30	Q9ZTQ4_MAIZE	Cell wall invertase	MAIZE

36	B6T871_MAIZE	Lactoylglutathione lyase	MAIZE
71	A0A096SXV7_MAIZE	alpha-mannosidase	MAIZE
84	B6SMK8_MAIZE	Inositol-1-monophosphatase	MAIZE
107	B6T9E1_MAIZE	Xyloglucan endotransglucosylase/hydrolase	MAIZE
113	K7VTU7_MAIZE	Uncharacterized protein	MAIZE
117	B8A306_MAIZE	phosphoglycerate mutase1	MAIZE
122	B4FS87_MAIZE	glyceraldehyde-3-phosphate dehydrogenase 2, cytosolic	MAIZE
125	B6SIG5_MAIZE	triosephosphate isomerase, cytosolic	MAIZE
139	B6UAK0_MAIZE	6-phosphogluconolactonase	MAIZE
**Lipid transport and metabolism (I)**			
111	P93852_MAIZE	Cycloartenol-C-24-methyltransferase 1	MAIZE
124	B6THH7_MAIZE	isoamyl acetate-hydrolyzing esterase	MAIZE
**Translation, ribosomal structure and biogenesis (J)**			
66	B4FL64_MAIZE	Ribosomal protein L19	MAIZE
73	B4FGK8_MAIZE	Eukaryotic translation initiation factor 2 alpha subunit	MAIZE
93	B6SX73_MAIZE	60S ribosomal protein L35	MAIZE
96	B6UHJ9_MAIZE	60S ribosomal protein L23a	MAIZE
97	B4FSQ4_MAIZE	40S ribosomal protein SA	MAIZE
114	Q9ZQX9_MAIZE	40S ribosomal protein S27	MAIZE
121	K7TNP5_MAIZE	eukaryotic translation initiation factor 3 subunit 3	MAIZE
146	B6T9H2_MAIZE	eukaryotic translation initiation factor 1A	MAIZE
34	B6STY9_MAIZE	Salt tolerance protein	MAIZE
38	B6U9I3_MAIZE	Eukaryotic translation initiation factor 3 subunit J	MAIZE
42	K7UTW6_MAIZE	Plasminogen activator inhibitor 1 RNA-binding protein	MAIZE
**Transcription (K)**			
54	K7V5M7_MAIZE	Uncharacterized protein	MAIZE
106	B6SIF0_MAIZE	Glycine-rich RNA-binding protein 2	MAIZE
**Transcription, Replication, recombination and repair (L)**			
72	A0A096PME5_MAIZE	Uncharacterized protein	MAIZE
**Cell wall/membrane/envelope biogenesis (M)**			
13	Q4U168_MAIZE	Glucose-1-phosphate adenylyltransferase	MAIZE
39	B4G1C2_MAIZE	Uncharacterized protein	MAIZE
52	SPSA_MAIZE	Sucrose-phosphate synthase	MAIZE
64	Q7XZQ2_MAIZE	UDP-glucose-4-epimerase	MAIZE

69	B6TXJ8_MAIZE	glycoside hydrolase, family 28	MAIZE
98	B6TA79_MAIZE	Cell division control protein 2	MAIZE
115	B6UD26_MAIZE	O-methyltransferase ZRP4	MAIZE
131	GLGL1_MAIZE	glucose-1-phosphate adenylyltransferase large subunit 1	MAIZE
144	B6SIF5_MAIZE	translationally-controlled tumor protein	MAIZE
145	B6SIF5_MAIZE	translationally-controlled tumor protein	MAIZE
**Posttranslational modification, protein turnover, chaperones (O)**			
4	Q41815_MAIZE	Heat shock protein 26	MAIZE
6	C6F1K2_ORYSJ	Heat shock protein 70	Oryza sativa subsp. japonica
9	B6TLK8_MAIZE	17.4 kDa class I heat shock protein 3	MAIZE
16	Q9S822_MAIZE	Heat shock protein 101	MAIZE
21	C0P5U8_MAIZE	Glutathione S-transferase	MAIZE
24	B6TSQ9_MAIZE	Aspartic proteinase oryzasin-1	MAIZE
28	B6SJE9_MAIZE	17.5 kDa class II heat shock protein	MAIZE
31	K7V5N4_MAIZE	PREDICTED: aspartic proteinase nepenthesin-1-like	MAIZE
35	B8A3K0_MAIZE	GST6 protein	MAIZE
51	B4F9E8_MAIZE	class II heat shock protein	MAIZE
57	A0A096TAP2_MAIZE	Uncharacterized protein	MAIZE
58	K7VD78_MAIZE	Putative chaperone clbp family protein	MAIZE
60	B4FRD1_MAIZE	Adaptin ear-binding coat-associated protein 1	MAIZE
61	B6THJ5_MAIZE	Phosphosulfolactate synthase-related protein	MAIZE
62	Q41886_MAIZE	27kDa storage protein, zein	MAIZE
82	B6SV64_MAIZE	Heat shock 70 kDa protein 4	MAIZE
99	A0A096QRB1_MAIZE	26S protease regulatory subunit 4 homolog	MAIZE
100	X4Z319_9POAL	Hsp70	Saccharum hybrid cultivar
103	B4F848_MAIZE	Chaperonin	MAIZE
109	A0A096QQ54_MAIZE	Uncharacterized protein	MAIZE
120	B4FNW7_MAIZE	activator of 90 kDa heat shock protein ATPase	MAIZE
130	B6THN2_MAIZE	ruBisCO large subunit-binding protein subunit beta	MAIZE
141	C0PFK3_MAIZE	proteasome subunit alpha type	MAIZE
143	Q9SM20_MAIZE	glutathione transferase III(b)	MAIZE
**Inorganic ion transport and metabolism (P)**			
45	B6TFC2_MAIZE	Selenium-binding protein	MAIZE
119	Q9XFZ3_ORYSJ	cytosolic monodehydroascorbate reductase	Oryza sativa subsp. japonica (Rice)

**Secondary metabolites biosynthesis, transport, and catabolism (Q)**			
17	B6UGU4_MAIZE	Steroleosin	MAIZE
25	B6SSB8_MAIZE	Transposon protein	MAIZE
59	B6UHC9_MAIZE	Putative uncharacterized protein	MAIZE
91	B4FU39_MAIZE	Uncharacterized protein	MAIZE
112	A0A096RS97_MAIZE	Uncharacterized protein	MAIZE
134	B6TF99_MAIZE	gibberellin 20 oxidase 2	MAIZE
138	B6U607_MAIZE	carbonyl reductase 1	MAIZE
**General function prediction only (R)**			
68	B6TRW7_MAIZE	Sigma factor sigB regulation protein rsbQ	MAIZE
**Signal transduction mechanisms(T)**			
46	B6TII3_MAIZE	ADP-ribosylation factor GTPase-activating protein 3	MAIZE
70	B6TA85_MAIZE	Protein binding protein	MAIZE
89	Q32SG1_MAIZE	Protein phosphatase 2A regulatory subunit B'	MAIZE
92	K7UYP8_MAIZE	Basic endochitinase A	MAIZE
127	A0A096TB53_MAIZE	USP family protein	MAIZE
**Intracellular trafficking, secretion, and vesicular transport (U)**			
33	B6UGN9_MAIZE	Oleosin	MAIZE
41	B6TTP4_MAIZE	Stress-inducible membrane pore protein	MAIZE
47	B6TKN6_MAIZE	Nonspecific lipid-transfer protein	MAIZE
74	A0A096QF11_MAIZE	Uncharacterized protein	MAIZE
75	M8CBN1_AEGTA	Importin-4	Aegilops tauschii
76	Q10EF5_ORYSJ	HEAT repeat family protein, expressed	Oryza sativa subsp. Japonica
80	B4FSU1_MAIZE	Transmembrane emp24 domain-containing protein 10	MAIZE
87	K7TNE7_MAIZE	Uncharacterized protein	MAIZE
90	B4FBP3_MAIZE	Signal recognition particle subunit SRP68	MAIZE
95	B9RET0_RICCO	Protein transport protein Sec24C	Sorghum bicolor
108	B4FTE1_MAIZE	Signal peptidase complex subunit 2	MAIZE
**Nuclear structure (Y)**			
142	B4FWD0_MAIZE	minor allergen Alt a 7	MAIZE
**Cytoskeleton (Z)**			
132	X1X262_ACYPI	actin related protein 1	Acyrthosiphon pisum
**Function unknown (S)**			
1	A3KLI1_MAIZE	RAB17 protein	MAIZE

2	B6SI37_MAIZE	Embryonic abundant protein	MAIZE
3	GLB1_MAIZE	Globulin-1 S allele	MAIZE
7	B8QY31_ZEAMP	Zeamatin-like protein	ZEAMP
8	B6SLF6_MAIZE	Antimicrobial peptide MBP-1	MAIZE
10	B6T8E4_MAIZE	Embryonic abundant protein 1	MAIZE
11	B6SQM6_MAIZE	Pathogeneis protein 10	MAIZE
12	K7W272_MAIZE	PREDICTED: globulin-1 S allele-like	MAIZE
14	A0A096TN72_MAIZE	PREDICTED: embryonic protein DC-8-like	MAIZE
15	A0A059PZ24_9POAL	Caleosin	Saccharum hybrid cultivar R570
18	B6UGS5_MAIZE	Putative uncharacterized protein	MAIZE
19	B4FFZ9_MAIZE	Lipoprotein	MAIZE
22	B6SK46_MAIZE	Cupin family protein	MAIZE
26	K7WCU4_MAIZE	Uncharacterized protein	MAIZE
27	Q94J20_ORYSJ	Lipoprotein-like	Oryza sativa subsp. Japonica
29	Q19VG6_MAIZE	Major latex protein 22	MAIZE
37	A0A096RTN1_MAIZE	PREDICTED: pathogenesis-related protein 1-like	MAIZE
40	B6SGN7_MAIZE	Embryonic protein DC-8	MAIZE
43	B6UDH8_MAIZE	Putative uncharacterized protein	MAIZE
48	B6TF92_MAIZE	Stress responsive protein	MAIZE
50	B8A0H4_MAIZE	Uncharacterized protein	MAIZE
56	C0HFQ1_MAIZE	Uncharacterized protein	MAIZE
63	A0A096TH62_MAIZE	Uncharacterized protein	MAIZE
65	A0A096QKY4_MAIZE	Uncharacterized protein	MAIZE
78	B4F922_MAIZE	Acyltransferase	MAIZE
79	B6U8P6_MAIZE	ABA-responsive protein	MAIZE
83	B4G1G1_MAIZE	Desiccation-related protein PCC13-62	MAIZE
85	A0A0A7LS12_SETIT	Up-frame shift 3 factor	Setaria italica
88	B4G1B0_MAIZE	Remorin	MAIZE
94	B4F957_MAIZE	pro-resilin	MAIZE
102	B6SMY5_MAIZE	Putative uncharacterized protein	MAIZE
105	B6T6D7_MAIZE	MIR-interacting saposin-like protein	MAIZE
123	C4J9Y2_MAIZE	uncharacterized protein LOC100502283	MAIZE
137	B8A230_MAIZE	unknown	MAIZE
148	ITRF_MAIZE	trypsin/factor XIIA inhibitor	MAIZE

Supplementary Table S5: KEGG pathway enrichment of identified DEPS.

Pathway	Seqs in Pathway	Enzyme	Ezyme ID	Seqs of Enzyme	Seqs
Chloroalkane and chloroalkene degradation	1	dehydrogenase (NAD+)	ec:1.2.1.3	1	tr|Q8S532|Q8S532_MAIZE
Phenylpropanoid biosynthesis	1	dehydrogenase	ec:1.1.1.195	1	tr|B4FU39|B4FU39_MAIZE
Glycolysis / Gluconeogenesis	1	dehydrogenase (NAD+)	ec:1.2.1.3	1	tr|Q8S532|Q8S532_MAIZE
Arginine and proline metabolism	1	dehydrogenase (NAD+)	ec:1.2.1.3	1	tr|Q8S532|Q8S532_MAIZE
Methane metabolism	1	dehydrogenase	ec:1.1.1.37	1	tr|E9NQE3|E9NQE3_MAIZE
Fatty acid degradation	1	dehydrogenase (NAD+)	ec:1.2.1.3	1	tr|Q8S532|Q8S532_MAIZE
Streptomycin biosynthesis	1	phosphatase	ec:3.1.3.25	1	tr|B6SMK8|B6SMK8_MAIZE
Purine metabolism	1	adenylpyrophosphatase	ec:3.6.1.3	1	tr|Q6R987|Q6R987_MAIZE
Purine metabolism	1	phosphatase	ec:3.6.1.15	1	tr|Q6R987|Q6R987_MAIZE
Steroid biosynthesis	1	24-C-methyltransferase	ec:2.1.1.41	1	tr|P93852|P93852_MAIZE
Limonene and pinene degradation	1	dehydrogenase (NAD+)	ec:1.2.1.3	1	tr|Q8S532|Q8S532_MAIZE
T cell receptor signaling pathway	1	phosphatase	ec:3.1.3.16	1	tr|K7V5M7|K7V5M7_MAIZE
Tryptophan metabolism	1	dehydrogenase (NAD+)	ec:1.2.1.3	1	tr|Q8S532|Q8S532_MAIZE
Arginine biosynthesis	1	acid amidohydrolase	ec:3.5.1.14	1	tr|C0PF66|C0PF66_MAIZE
Valine, leucine and isoleucine degradation	1	dehydrogenase (NAD+)	ec:1.2.1.3	1	tr|Q8S532|Q8S532_MAIZE
Thiamine metabolism	1	phosphatase	ec:3.6.1.15	1	tr|Q6R987|Q6R987_MAIZE
beta-Alanine metabolism	1	dehydrogenase (NAD+)	ec:1.2.1.3	1	tr|Q8S532|Q8S532_MAIZE
Glyoxylate and dicarboxylate metabolism	1	dehydrogenase	ec:1.1.1.37	1	tr|E9NQE3|E9NQE3_MAIZE
Lysine degradation	1	dehydrogenase (NAD+)	ec:1.2.1.3	1	tr|Q8S532|Q8S532_MAIZE
Ascorbate and aldarate metabolism	1	dehydrogenase (NAD+)	ec:1.2.1.3	1	tr|Q8S532|Q8S532_MAIZE
Citrate cycle (TCA cycle)	1	dehydrogenase	ec:1.1.1.37	1	tr|E9NQE3|E9NQE3_MAIZE
Phosphatidylinositol signaling system	1	phosphatase	ec:3.1.3.25	1	tr|B6SMK8|B6SMK8_MAIZE
Inositol phosphate metabolism	1	phosphatase	ec:3.1.3.25	1	tr|B6SMK8|B6SMK8_MAIZE
Glycerolipid metabolism	1	dehydrogenase (NAD+)	ec:1.2.1.3	1	tr|Q8S532|Q8S532_MAIZE
Cysteine and methionine metabolism	1	dehydrogenase	ec:1.1.1.37	1	tr|E9NQE3|E9NQE3_MAIZE
Carbon fixation pathways in prokaryotes	1	dehydrogenase	ec:1.1.1.37	1	tr|E9NQE3|E9NQE3_MAIZE

Aminobenzoate degradation	2	nitrophenyl phosphatase	ec:3.1.3.41	2	tr|B6SMK8|B6SMK8_MAIZE, tr|K7V5M7|K[illegible]
Pentose and glucuronate interconversions	2	dehydrogenase (NAD+)	ec:1.2.1.3	1	tr|Q8S532|Q8S532_MAIZE
Pentose and glucuronate interconversions	2	pectin depolymerase	ec:3.2.1.15	1	tr|B6TXJ8|B6TXJ8_MAIZE
Carbon fixation in photosynthetic organisms	2	dehydrogenase	ec:1.1.1.37	1	tr|E9NQE3|E9NQE3_MAIZE
Carbon fixation in photosynthetic organisms	2	dehydrogenase (decarboxylating)	ec:1.1.1.39	1	tr|Q5G1U0|Q5G1U0_MAIZE
Glutathione metabolism	2	transferase	ec:2.5.1.18	2	tr|B8A3K0|B8A3K0_MAIZE, tr|C0P5U8|C0P[illegible]
Drug metabolism - cytochrome P450	2	transferase	ec:2.5.1.18	2	tr|B8A3K0|B8A3K0_MAIZE, tr|C0P5U8|C0P[illegible]
Histidine metabolism	2	histidinol phosphate phosphatase	ec:3.1.3.15	1	tr|B6SMK8|B6SMK8_MAIZE
Histidine metabolism	2	dehydrogenase (NAD+)	ec:1.2.1.3	1	tr|Q8S532|Q8S532_MAIZE
Metabolism of xenobiotics by cytochrome P450	2	transferase	ec:2.5.1.18	2	tr|B8A3K0|B8A3K0_MAIZE, tr|C0P5U8|C0P[illegible]
Galactose metabolism	2	4-epimerase	ec:5.1.3.2	1	tr|Q7XZQ2|Q7XZQ2_MAIZE
Galactose metabolism	2	invertase	ec:3.2.1.26	1	tr|Q9ZTQ4|Q9ZTQ4_MAIZE
Starch and sucrose metabolism	2	invertase	ec:3.2.1.26	1	tr|Q9ZTQ4|Q9ZTQ4_MAIZE
Starch and sucrose metabolism	2	alpha-glucosidase	ec:3.2.1.48	1	tr|Q9ZTQ4|Q9ZTQ4_MAIZE
Starch and sucrose metabolism	2	pectin depolymerase	ec:3.2.1.15	1	tr|B6TXJ8|B6TXJ8_MAIZE
Pyruvate metabolism	3	dehydrogenase	ec:1.1.1.37	1	tr|E9NQE3|E9NQE3_MAIZE
Pyruvate metabolism	3	dehydrogenase (decarboxylating)	ec:1.1.1.39	1	tr|Q5G1U0|Q5G1U0_MAIZE
Pyruvate metabolism	3	dehydrogenase oxaloacetate-decarboxylating	ec:1.1.1.38	1	tr|Q5G1U0|Q5G1U0_MAIZE
Pyruvate metabolism	3	dehydrogenase (NAD+)	ec:1.2.1.3	1	tr|Q8S532|Q8S532_MAIZE
Amino sugar and nucleotide sugar metabolism	4	4-epimerase	ec:5.1.3.2	1	tr|Q7XZQ2|Q7XZQ2_MAIZE
Amino sugar and nucleotide sugar metabolism	4	chitodextrinase	ec:3.2.1.14	3	tr|B4G1C2|B4G1C2_MAIZE, tr|C0P451|C0P[illegible]
Biosynthesis of antibiotics	5	dehydrogenase	ec:1.1.1.37	1	tr|E9NQE3|E9NQE3_MAIZE
Biosynthesis of antibiotics	5	phosphatase	ec:3.1.3.25	1	tr|B6SMK8|B6SMK8_MAIZE
Biosynthesis of antibiotics	5	24-C-methyltransferase	ec:2.1.1.41	1	tr|P93852|P93852_MAIZE
Biosynthesis of antibiotics	5	dehydrogenase (NAD+)	ec:1.2.1.3	1	tr|Q8S532|Q8S532_MAIZE
Biosynthesis of antibiotics	5	acid amidohydrolase	ec:3.5.1.14	1	tr|C0PF66|C0PF66_MAIZE

Supplementary Table S6: Primers used in qRT-PCR

Protein No.	Primer pairs (5'--3')
1	F: 5’-ACCAGTACGGCAACCCAGTC-3’<br>R: 5’-TTAAACACGAAACCCACGGA-3’
2	F: 5’- TACAGCGAGATGGGGAAGAA-3’<br>R: 5’- CGTGAAACAGGCTACCAAACTAC-3’
3	F: 5’- CGTTCGTTCAAGATCGCCTAC-3’<br>R: 5’- CACCTTCTCGTTCCTGTCGG-3’
4	F: 5’- CGCAGGAGAACAGGGACAA-3’<br>R: 5’- GGCGACATCGGGTCAACTA-3’
5	F: 5’- ACAAGAAGCCGACCCATTCC-3’<br>R: 5’- TCCTCGACTTTTGCCCACC-3’
6	F: 5’- GCGAGCGAATCAGAAGAGCA-3’<br>R: 5’-TCAAAGACGGTGTTGGTGGG-3’
7	F: 5’- ACTTCTTCGACATCTCCCTCATC-3’<br>R: 5’- TGCTGGTGGCGTCATCCTT-3’
8	F: 5’- GCCATCTCCTCACCCTCGTT-3’<br>R: 5’-CCTTTCCGTACTGCCACTCG-3’
9	F: 5’- GCCAAGAAGCCTGAAGTGAAG-3’<br>R: 5’- AAGTAGACCCAACATACGCAAAG-3’
10	F: 5’- AGGAAAGCAGGGAGGAGCTG-3’<br>R: 5’- GGACTTGGTCCTGAACTTGGA-3’
11	F: 5’- TGCGTCGCCAAGGTCAA-3’<br>R: 5’- CCAACTCACAAACGTCGGTCT-3’
12	F: 5’- AACTTTGAGCAGGGGAGCAT-3’<br>R: 5’- GCGGTGTCTTGGCAGACTTG-3’
13	F: 5’- GACACGAACTCAGGTCCTCACC-3’<br>R: 5’- CCGCCCAAAATGATAGCAGAT-3’
14	F: 5’- CACTAGCGTCGAGCCATTACA-3’<br>R: 5’- TCTCCTGATACGGGACCACAG-3’
15	F: 5’- GACCTCTACGGCTGGATTGC-3’<br>R: 5’- GTTCACTTGGCTTCCATTTCAT-3’
16	F: 5’- TGAGACGGGGATGAAGTCG-3’<br>R: 5’- ACAAACAGCAGCGTTACAAGAA-3’

17	F: 5'- GCGTCCATCTGGCAAGTGT-3'<br>R: 5'- TCCATCCTCAGGGTGTCGTA-3'
86	F: 5'- CTTGTCTATTGCCCTTCGGAGTT-3'<br>R: 5'- CCGCCAAGATTGATGCTAAAA-3'
87	F: 5'- CCTCACTACAGGGAACAGCG-3'<br>R: 5'- CTTGAAGATGATGGAAGTATGGG-3'
88	F: 5'- ACACCGAGCAGAAAGTGAGAAAC-3'<br>R: 5'- TGTCAACAACAACGAAAACAACAG-3'
89	F: 5'- GCCGCTTCAGCATTTCG-3'<br>R: 5'- GCCTCTTTATCTCCTTCTCCTTTAT-3'
90	F: 5'- GTACAAACGCAAAACAACGC-3'<br>R: 5'- CAATCATAGGCTCCACTTCG-3'
91	F: 5'- CCCCGTTCCATTTCATTATCC-3'<br>R: 5'- CGTGACATCGTCGTCTTTAGG-3'
92	F: 5'- ATCGGCTACGGGAGCAATCT-3'<br>R: 5'- TTCAAACCGACGAAGCAACAC-3'
93	F: 5'- TCTCGCAGAAGCAGAAGTCG-3'<br>R: 5'- CAGCAAATGTCCATACAAACCA-3'
94	F: 5'- GGCGGACGACTTCGATGAGT-3'<br>R: 5'- CGAGCACGGGTAGCAGGTTT-3'
95	F: 5'- TCTACTGGCGGAAAACTTCTTG-3'<br>R: 5'- CCACCTGTAGTCTGAGGCACAA-3'
96	F: 5'- ATCAGCACTACCGGAAGGAAC-3'<br>R: 5'- GCATCATAGTCGGGAGTCAGC-3'
97	F: 5'- CCAGCGTGCTGTCCTCAAGT-3'<br>R: 5'-AAGGCAATGGTCGGGATGTT-3'
98	F: 5'- TGGTCTGTGGGCTGTATCTTTG-3'<br>R: 5'- TCTTTTGCTTGGCTCGTATCG-3'
99	F: 5'- TGTGGCTAACTCAACATCTGCG-3'<br>R: 5'- CCAACAGCGTCAATCTCATCAATAA-3'
100	F: 5'- GTTCAGGGGACAACAAACCTTATG-3'<br>R: 5'- AACCAGCCTACAGCGGAGAAA-3'
101	F: 5'- AATACCGATACCTGTCAGATTCCA-3'<br>R: 5'- GTGTTCAAGCAGTGCGTCCA-3'

102	F: 5’- GACGAGGCCGAGAAGGAGAT-3’<br>R: 5’- TGACTGACGAAAGGAAACTAATACG-3’
103	F: 5’- CTCTGGGAGATAGGGTGCTTGT-3’<br>R: 5’- ATTTTCTTGTCCCCGATGGTC-3’
104	F: 5’- CTAGCTGACCAATTCGACACGA-3’<br>R: 5’- AGGAGGAGCAAGACGAGGGA-3’
105	F: 5’- CATCTGTAAAACGACCACCCC-3’<br>R: 5’- ATGACCTTCCCCTCCCTCTG-3’
106	F: 5’- TCCTTTTCCTTTCATAAACCCC-3’<br>R: 5’- TCACCCTCGCTTCAGTAACCT-3’
107	F: 5’- AGCTATCCAGTGGAGACGAAGAT-3’<br>R: 5’- CTGCGACGACAGGTAGAAGG-3’
108	F: 5’- AAAGGAAGGAGTTCTTGTGGAGG -3’<br>R: 5’- GTTTCTAGGATTCGGACGGATAGT-3’
109	F: 5’- ATTTTATGCCAGCGAGAAGTATCC-3’<br>R: 5’- CATCCTCCAGCCATCAGACAAC-3’
110	F: 5’- CATCCTCCAGCCATCAGACAAC-3’<br>R: 5’- AGACACCATCACCAGTCCAAGC-3’
111	F: 5’- TTTTAGATGTGGGCTGTGGAATAG-3’<br>R: 5’- TCTCCTTGTAGCAACCAACTGGA-3’
112	F: 5’- GTCCTCAACGTCAACCACTACC-3’<br>R: 5’- TCAACGCTCTTCAGCAATCC-3’
113	F: 5’- GCCTCCGTTCTCGTCAATG-3’<br>R: 5’- TCCAAGCCGTTCCAGTTGC-3’
114	F: 5’- CCAGTCGCCCAACTCCTTCT-3’<br>R: 5’- TCAGTGAGCCTTGCCTTTCCT-3’
115	F: 5’-GGGAGTTCAGGGACTTGTTCG-3’<br>R: 5’- GGTGGCAATGATCTTGGGAG-3’
116	F: 5’-TCTTCGCCGAGCAGTTTATC-3’<br>R: 5’- TTCGCCACTTGTTCCATACC-3’
117	F: 5’- GGTGTCCACTCCCGTCTTGA-3’<br>R: 5’- TTGAATTTGTAGGGTGCTTCTCC-3’
118	F: 5’- CATCTCGGGACCAACTAACCAA-3’<br>R: 5’- ACACCTGTGAACCCAGCGTATT-3’
119	F: 5’- CAAGTTCGGCTCGTACTGGATT-3’<br>R: 5’- GAACCTTCACGATGACACGGA-3’
120	F: 5’- CAGGAAGGAAAACTGATTGCG-3’

	R: 5’- AACTGGGTGAGAAACATGGATTG-3’
121	F: 5’- AGGAGAACATGGCAAGAAAAGC-3’<br>R: 5’- TGTCGAAATTCTGACCAGCAAC-3’
122	F: 5’-GGCTGGTGCTGAGTATGTCGTG-3’<br>R: 5’- TGAGGGTCCATCAACAGTCTTCT-3’
123	F: 5’- CTACGAGAACCCCAAGCACAA-3’<br>R: 5’- GCCACTATGTTACACCACCATCC-3’
124	F: 5’- CTACGAGAACCCCAAGCACAA-3’<br>R: 5’- GCCACTATGTTACACCACCATCC-3’
125	F: 5’- CCTGGGTCATTCTTGGACACTC-3’<br>R: 5’- CCAATAGCCCAAACTGGTTCATA-3’
126	F: 5’- TCCGTGACACCATCGACCTC-3’<br>R: 5’- GGCAACTTCTTCGGCTTCCT-3’
127	F: 5’- TCAAGGAGCCCGACTACGAG-3’<br>R: 5’- CAGGATGTCCAAGGTTTCTATGTC-3’
